# Hexose phosphorylation for a non-enzymatic glycolysis and pentose phosphate pathway on early Earth

**DOI:** 10.1038/s41598-023-50743-8

**Published:** 2024-01-02

**Authors:** Yuta Hirakawa, Takeshi Kakegawa, Yoshihiro Furukawa

**Affiliations:** Department of Earth Science, Tohoku University, 6-3, Aza-Aoba, Aramaki, Aoba-Ku, Sendai, 980-8578 Japan

**Keywords:** Astrobiology, Geochemistry, Origin of life

## Abstract

Glycolysis and pentose phosphate pathways play essential roles in cellular processes and are assumed to be among the most ancient metabolic pathways. Non-enzymatic metabolism-like reactions might have occurred on the prebiotic Earth and been inherited by the biological reactions. Previous research has identified a part of the non-enzymatic glycolysis and the non-enzymatic pentose phosphate pathway from glucose 6-phosphate and 6-phosphogluconate, which are intermediates of these reactions. However, how these phosphorylated molecules were formed on the prebiotic Earth remains unclear. Herein, we demonstrate the synthesis of glucose and gluconate from simple aldehydes in alkaline solutions and the formation of glucose 6-phosphate and 6-phosphogluconate with borate using thermal evaporation. These results imply that the initial stages of glycolysis-like and pentose phosphate pathway-like reactions were achieved in borate-rich evaporative environments on prebiotic Earth, suggesting that non-enzymatic metabolism provided biomolecules and their precursors on prebiotic Earth.

## Introduction

**Figure 1:**
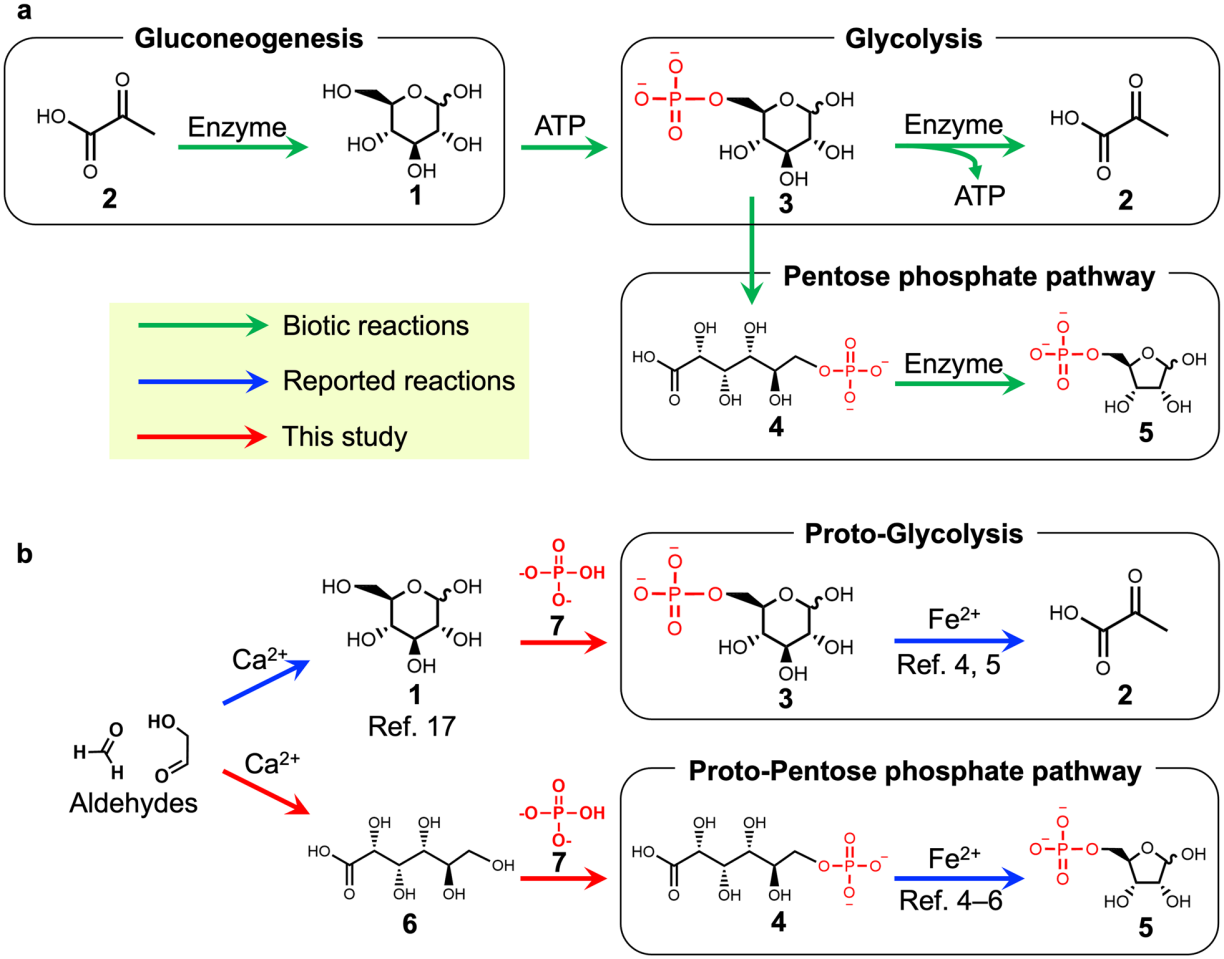
Comparison of current metabolic reactions and possible prebiotic reactions. (**a**) The metabolic reactions in the current life. (**b**) Possible prebiotic formation of glucose 6-phosphate 3 and 6-phosphogluconate 4 investigated in the present study and possible non-enzymatic metabolism-like reactions reported previously^4–6^.

Glycolysis and pentose phosphate pathways yield many molecules, including the precursors of the building blocks of life, an intermediate of the TCA and rTCA cycles, and ATP, which has high-energy phosphate bonds. Glycolysis employs glucose **1** as the starting molecule and breaks it down into pyruvate **2**, coupled with the formation of ATP (Fig. 1a). Pentose phosphate pathways start from glucose 6-phosphate **3**, which is oxidized into 6-phosphogluconate **4** and subsequently forms many kinds of sugar phosphates, including ribose 5-phosphate **5**, a necessary precursor of nucleotides and RNA for genetic processes (Fig. 1a). These metabolic pathways are ubiquitous in almost all living organisms; thus, they are widely believed to be some of the most ancient metabolisms^1–3^.

Some of these ancient metabolisms could have already emerged during prebiotic Earth as metabolism-like reactions promoted by abiotic catalysts. These processes could have then been inherited by the metabolic pathways of ancient life forms^4–14^. While current life forms employ sophisticated enzymes to catalyze metabolic pathways, such reactions in ancient life forms would have been catalyzed by more primitive catalysts such as metal ions^4–6,12–14^. Ralser et al. investigated non-enzymatic glycolysis and non-enzymatic pentose phosphate pathway and showed that Fe^2+^ can catalyze many reactions of glycolysis and pentose phosphate pathway from glucose 6-phosphate **3** and 6-phosphogluconate **4**, supplying various biomolecules such as pyruvate **2** and ribose 5-phosphate **5**^4–6^. However, how glucose 6-phosphate **3** and 6-phosphogluconate **4** were abiotically formed on prebiotic Earth remains unclear (Fig. 1b).

The formation of sugars, including glucose **1**, during prebiotic Earth by the formose reaction has been suggested in previous studies^15–17^. The formose reaction is generally known to form sugars from simple aldehydes supplied by photochemical reactions of atmospheric CO_2_ and H_2_O^18^. Identification of pentose sugars in meteorites in previous studies supports the formation of sugars in abiotic geological environments^19^. In contrast, the formation of sugar acids, such as gluconate **6**, which has been detected in meteorites, has not been well investigated^20,21^. Several previous studies have investigated the formation of glucose phosphates^22–24^. The classical reaction employed cyanogen as a condensation agent, resulting in the formation of glucose 1-phosphate. This was followed by an intermolecular phosphate transfer process, leading to the creation of  glucose 1,6-bisphosphate and glucose 6-phosphate **3**^22^. However, the formation of cyanogen on the prebiotic Earth lacks support from geochemical evidence. An alternative method attempted to generate glucose phosphates, although the phosphorylation sites remianed undetermained. This method involved incubating glucose and phosphate at high temperatures in the presence of some clay minerals, simulating acidic hydrothermal conditions^23^. More recently, the selective formation of glucose 1-phosphate from glucose **1** in microdroplets was reported^24^. Thus, the formation of glucose 6-phosphate **3** and 6-phosphogluconate **4** on the prebiotic Earth still present uncertainties and require further investigation.

We recently reported that pentose phosphate can be formed from pentose and orthophosphate **7** with the help of borate **8** and urea **9**^25,26^. Borate **8** is known to stabilize sugars^27,28^ and urea **9** functions as a phosphorylation catalyst in prebiotic chemistry^29,30^. The most common phosphate mineral, apatite, is generally insoluble. However, this mineral would have released phosphate **7** into the early ocean with the help of some coexisting anions, such as carbonate or formate^26^. These anions can remove Ca by combining with Ca^2+^ to form precipitates (Ca carbonate minerals) or complex (formic acid chelate) and solubilizing phosphate **7** from apatite. The early atmosphere is generally assumed to be rich in CO_2_, indicating that the early hydrosphere contained abundant carbonate. Further, it has been reported that a eutectic solution containing urea can mobilize various phosphate minerals, including apatite^30^. Another potential source of phosphorus is a phosphide mineral, such as schreibersite. This mineral was found in meteorites, suggesting its supply to the prebiotic Earth^31^. Phosphide species can transform into phosphite and phosphate by reacting with water, providing soluble phosphorus compounds to the early environments^31^. A borosilicate mineral, tourmaline, has been found in early Archean (i.e., ~ 3.8 Ga) metasedimentary rocks^32–34^. The boron isotope compositions of tourmaline suggest that the boron was concentrated in isolated basins on the early Archean Earth^34,35^. In addition, early Archean pseudomorphic concretions after evaporites suggest the existence of evaporative basins on the early Archean Earth (i.e., ~ 3.22 Ga)^36^. Although Hadean rocks are not available for investigation, Hadean minerals indicate the formation of granitic rocks, which are the typical rocks that form continental crust on the present Earth, suggesting the emergence of land on Hadean Earth^37^. The land area during this period would have been small, but this is consistent with the boron-rich evaporative basin that has been suggested as an early Archean geological setting^32,34,35^. Many investigations on chemical evolution simulating evaporative environments have been conducted^25,26,30,38,39^. Urea **9** would have been formed through the hydrolysis of a cyan molecule formed via meteorite impacts^40,41^. The pentose phosphorylation may be also able to be applied to the phosphorylation of other sugars and sugar acids. However, it is unclear whether phosphorylation occurs at 6-hydroxyl of glucose **1** and gluconate **6** because these molecules have five hydroxyls that can react with phosphate **7** equally. Thus, we investigated whether glucose **1** and gluconate **6** are formed simultaneously by a formose-like reaction, and whether these molecules can be regioselectively phosphorylated to form glucose 6-phosphate **3** and 6-phosphogluconate **4** under prebiotically plausible conditions.

## Results

**Figure 2:**
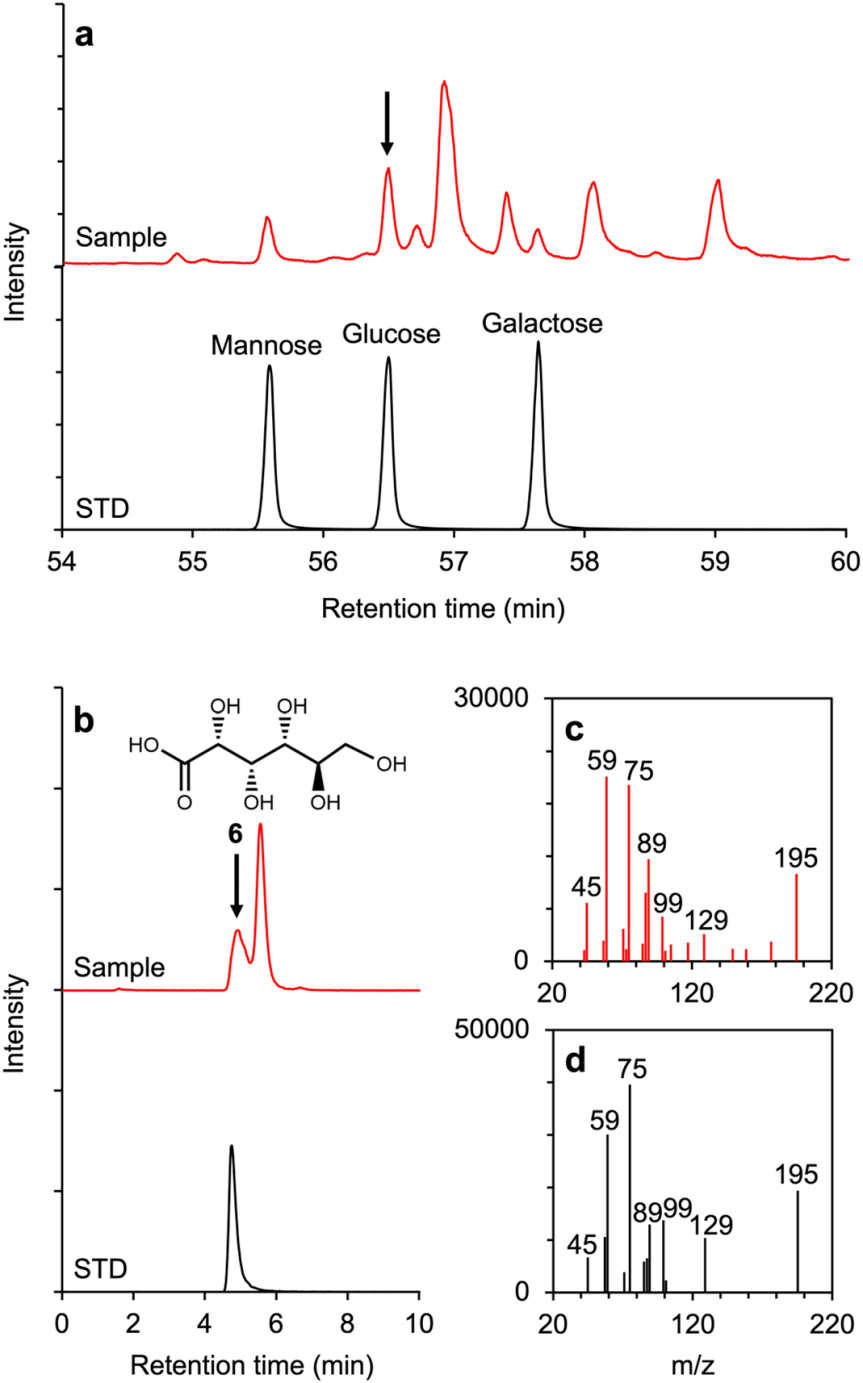
Identification of sugars and gluconic acid in the formose-like reaction products. (**a**) GC–MS single ion chromatograms of the derivatized product of the formose-like reaction experiment and the derivatized standard (m/z: 145). (**b**) LC–MS/MS multiple reaction monitoring (MRM) chromatograms of the formose-like reaction product and gluconic acid (m/z: 195 > 75) (**c**) Product ion spectrum of the experimental product shown with an arrow in (**b**). (**d**) Product ion spectrum of the gluconic acid standard.

Here, we demonstrate the abiotic synthesis of glucose **1** and gluconate **6** and their selective phosphorylation to glucose 6-phosphate **3** and 6-phosphogluconate **4**. First, we performed formose-like reaction experiments with aqueous solutions containing 100 mM formaldehyde, 10 mM glycolaldehyde, and a catalyst for the formose-like reaction, 10 mM Ca(OH)_2_, at 95 °C for 72 h. The products were analyzed by gas chromatography-mass spectrometry (GC–MS) and liquid chromatography-tandem mass spectrometry (LC–MS/MS) for sugar identification after derivatization and sugar acid identification, respectively. The analysis showed the formation of many kinds of sugars and sugar acids, including glucose **1** and gluconate **6** (Fig. 2 and Fig. S1). The concentrations of glucose **1** and gluconate **6** in the experimental products were 48 and 36 µM (0.048 and 0.036 mol%, based on the formaldehyde concentration in the starting material), respectively.

**Figure 3:**
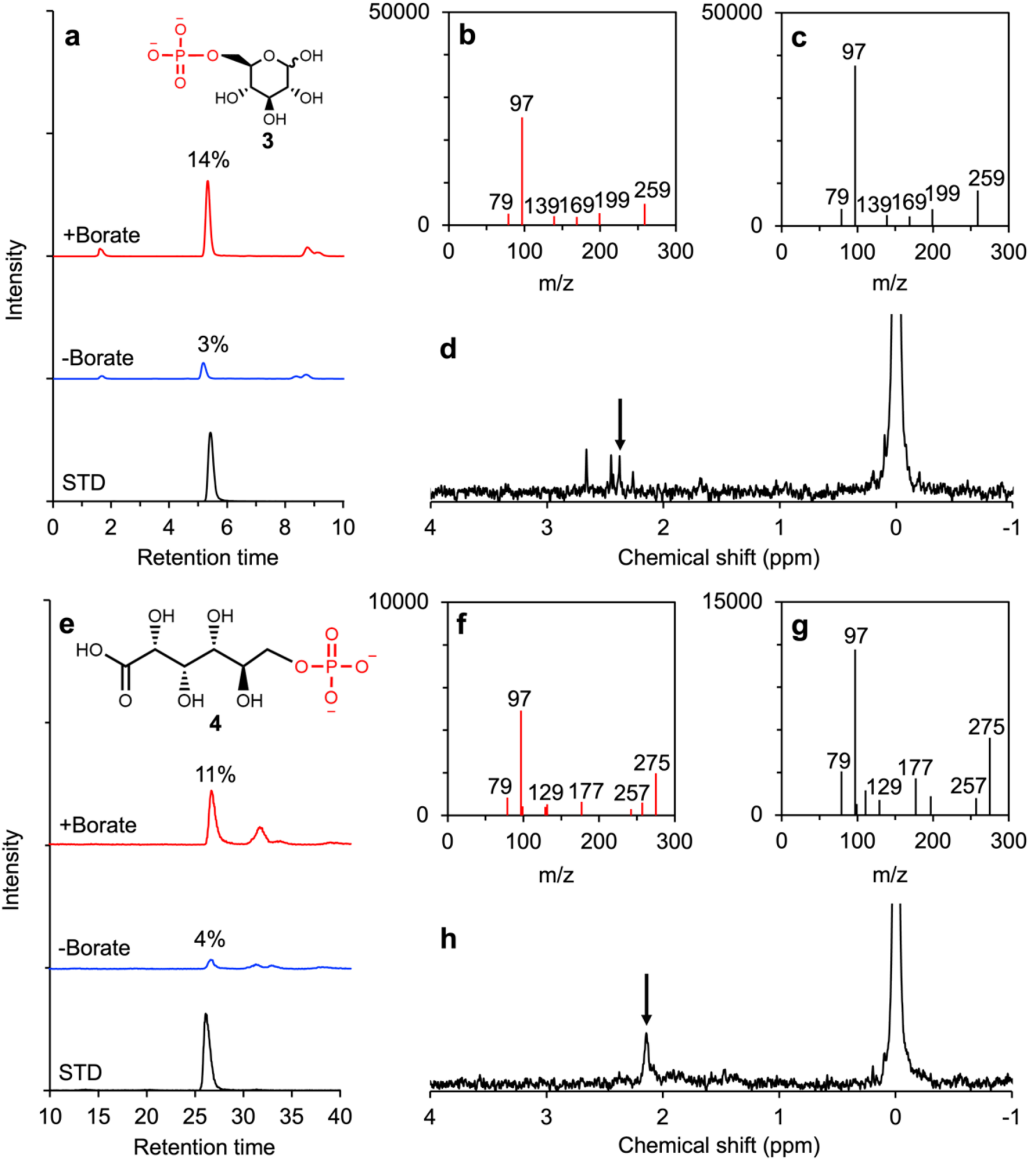
Identification of glucose 6-phosphate and 6-phosphogluconate by LC–MS/MS and NMR. (**a**) LC–MS/MS MRM chromatograms of the experimental product of glucose phosphorylation at 80 °C and glucose 6-phosphate standard (m/z: 259 > 97). The yields represent the average of triplicated experiments. (**b**) Product ion spectrum of the experimental product of glucose phosphorylation in the borate-added product at 80 °C. The mass signals of m/z of 259 and 97 correspond to glucose-phosphate and phosphate, respectively. (**c**) Product ion spectrum of the glucose 6-phosphate standard. (**d**) ^31^P-NMR spectrum of the experimental product of glucose phosphorylation at 80 °C (see also Fig. S4). (**e**) LC–MS/MS MRM chromatograms of the experimental product of gluconate phosphorylation at 80 °C and 6- phosphogluconate standard (m/z: 275 > 97). The yields represent the average of triplicated experiments. (**f**) Product ion spectrum of the experimental product of gluconate phosphorylation in the borate-added product at 80 °C. The mass signals of m/z of 275 and 97 correspond to phosphogluconate and phosphate, respectively. (**g**) Product ion spectrum of the 6-phosphogluconate standard. (**h**) ^31^P-NMR spectrum of the experimental product of gluconate phosphorylation at 80 °C (see also Fig. S5).

Furthermore, we conducted thermal evaporation of glucose **1** and gluconate **6** solutions with phosphate to induce phosphorylation. Aqueous solutions containing 160 mM Na_2_HPO_4_ and 20 mM glucose or 20 mM sodium gluconate were dried at 80 or 95 °C for 24 h in the presence of 40 mM borate **8** and 800 mM urea **9**. The products were hydrolyzed using an acidic solution and identified using LC–MS/MS. The analysis indicated the formation of glucose 6-phosphate **3** and 6-phosphogluconate **4** based on their LC retention time and fragmentation spectra identical to those of the standards (Fig. 3, Figs. S2–S5). This analysis also excludes the possibility of the formation of 2-, 3-, and 4-phosphate of glucose (Fig. S5). Before the acid-hydrolysis, glucose phosphate was most likely present as ureido-glucose phosphate, as shown by an LC–MS/MS analysis (Fig. S6).

The formation of glucose phosphate and phosphogluconate was also confirmed by ^31^P-nuclear magnetic resonance (NMR) analysis of the product otained from thermal evaporation of an aqueous solution containing 200 mM glucose or gluconate, 400 mM borate, 1.6 M K_2_HPO_4_, and 4 M urea, followed by acid hydrolysis (Fig. 3, Figs. S7, and S8). This experiment was conducted to obtain a concentrated glucose phosphate or phosphogluconate for the NMR experiments. ^31^P-NMR spectra showed peaks from phosphorylated sugars or sugar acids, orthophosphate, and unknown peaks considered polyphosphoric compounds (Fig. S9).

To obtain a more detailed structure of glucose phosphate, we compared the ^13^C-NMR spectra of prepared ureido-glucose and its phosphorylated product (Figs. S10 and S11). The ureido-glucose was prepared from the thermal evaporation of aqueous solution containing 200 mM glucose, 400 mM borate, and 800 mM. The phosphorylated ureido-glucose was obtained from the thermal evaporation of an aqueous solution containing 200 mM glucose, 400 mM borate, 1.6 M K_2_HPO_4_, and 4 M urea without acid hydrolysis. The ^13^C-NMR spectrum of the product of the phosphorylation experiment shows almost identical peaks that were observed in the ^13^C-NMR spectrum of prepared ureido-glucose, except for an additional peak around ~ 62.5 ppm, which is close to the peaks of 6-carbon of ureido-glucose, indicating that the 6-carbon was selectively phosphorylated (Fig. S11). The ^31^P-NMR of the product phosphate showed almost the same chemical shift as that of the standard of glucose 6-phosphate **3** (Fig. S8d).

**Figure 4:**
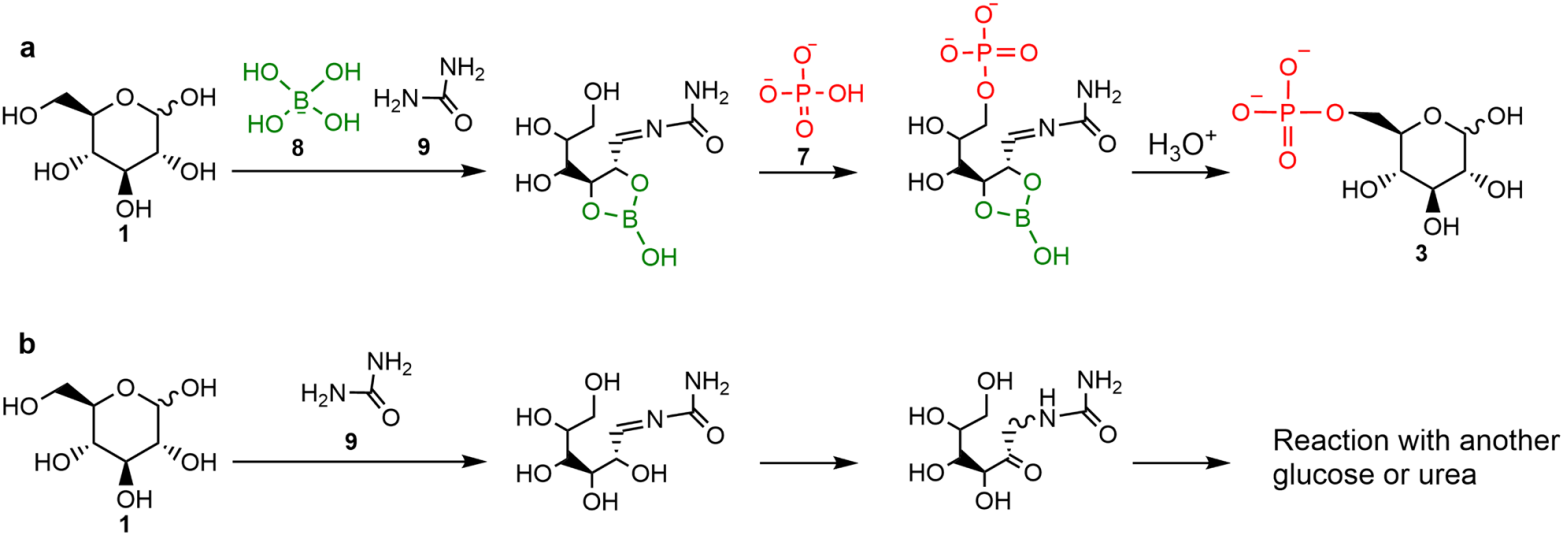
Borate-guided regioselective phosphorylation of glucose at 6-hydroxyl position. (**a**) Possible glucose phosphorylation pathway with borate. (**b**) Possible reaction pathway without borate.

The yields of glucose 6-phosphate **3** and 6-phosphogluconate **4** were 14.2 ± 1.1 mol% and 11 ± 4.0 mol% on average at 80 °C, and 12.1 ± 0.8 mol% and 16.8 ± 1.1 mol% on average at 95 °C, respectively (n = 3). The product yields were higher in the presence of borate **8** compared to its absence in both experiments (Fig. 3). The residual amounts of glucose **1** and gluconate **6** were 5.9 ± 0.2 mol% and 15.7 ± 3.7 mol%, respectively (Figs. S12 and S13). To investigate the effects of borate on the structure of glucose, we compared the ^1^H-NMR and ^13^C-NMR spectra of glucose with urea in the presence and absence of borate. The products were obtained by the condensation of 200 mM glucose and 800 mM urea to form ureido-glucose in the presence and absence of 400 mM borate, those of which were analyzed with ^1^H-NMR and ^13^C-NMR (Figs. S14–S16). The number of peaks was substantially limited in the presence of borate **8**, showing that borate **8** constrains the structure of ureido-glucose (Fig. 4a). We also confirmed the formation of ureido-glucose combined with borate. A thermal evaporation product of 100 µL of aqueous solution containing 100 mM glucose, 100 mM urea, and 800 mM borate was dissolved in 1 mL water, followed by a direct infusion MS spectrometry analysis. The spectrum showed the formation of ureido-glucoses combined with one or two borates (Fig. S17).

We further performed the phosphorylation experiment in the presence of glucose, gluconate, and Ca^2+^ to be consistent with the experimental condition of the formose-like reaction described above. The experimental solution containing 20 mM glucose, 20 mM gluconate, 40 mM borate, 160 mM Na_2_HPO_4_, 800 mM urea, and 10 mM CaCl_2_ was dried at 80 °C for 24 h, followed by the acid hydrolysis and LC–MS/MS analysis. The analysis showed that both glucose 6-phosphate **3** and 6-phosphogluconate **4** were formed in the same pot with considerable yields, 13.8 ± 0.7% and 11.7 ± 0.3%, respectively (Figs. S18 and S19).

The thermal evaporation experiments were also conducted using hexoses (mannose **10**, galactose **11**, and fructose **12**) instead of glucose **1**. The formation of mannose phosphate and galactose phosphate was confirmed (9.1 ± 0.9 mol% and 30.3 ± 1.5 mol% yield on average, respectively; n = 3). These yields were calculated using the calibration curve of glucose 6-phosphate **3** based on the assumption that every phosphorylated product has almost the same peak area following previously reported analysis of sugar phosphates^25^. Even for these sugars, the amounts of products were higher in the presence of borate **8** than in its absence (Figs. S20 and S21). In contrast, fructose phosphate formation was not observed (i.e., less than 1 mol%). The residual rates of each sugar were 2–15 mol% in the presence of borate **8** and 1–2 mol% in its absence (Figs. S22 and S23).

## Discussion

Formose-like reactions form various sugars and sugar-related molecules via the polymerization of formaldehyde and glycolaldehyde. Glucose formation via a formose-like reaction has been reported previously^17^. In the present study, we confirmed the formation of aldohexoses, including glucose **3** and ketohexoses, including fructose **12** (Fig. 2 and Fig. S1). Fructose formation has not been reported in previous studies on prebiotic reactions.

We also confirmed the formation of sugar acids, including gluconate **6**, in the reaction products. The carbon structures of the sugar acids were formed via the polymerization of formaldehyde and glycolaldehyde, similar to sugars. In addition, sugar acids have a carboxyl at 1′-position instead of aldehyde. Thus, oxidation is necessary for gluconate **6** after hexose formation. Sugar acids were synthesized via a combination of formose reaction and cross-Cannizzaro reactions. The cross-Cannizzaro reaction oxidizes an aldehyde via the reduction of another aldehyde^42^.

Among the different phosphorylation sites in glucose **1** and gluconate **6**, the 6-hydroxyl group was preferentially phosphorylated, forming glucose 6-phosphate **3** and 6-phosphogluconate **4**, which are biomolecules used in glycolysis and pentose phosphate pathways in modern cells (Fig. 3, Figs. S7, and S8). The primary phosphorylation site for glucose is generally 1-aldehyde^22–24^. However, in the present reaction, urea **9** reacted with glucose **1** at 1-aldehyde to form ureido-glucose. Borate **8** can combine the remaining hydroxyls (Fig. 4 and Fig. S24). Ureido-glucose that combines borate **8** at 2,3-hydroxyls tends to have improved stability, avoiding isomerization (Fig. S24). This improvement in stability could increase the yields of glucose 6-phosphate and remaining glucose as seen in this study.

In contrast to glucose **1**, gluconate **6** has a carboxyl group instead of an aldehyde. Gluconate **6** does not combine with urea **9** at 1 position since carboxyl carbons generally have lower electrophilicity than formyl carbons. Four of the five hydroxyl groups of gluconate **6** can react with borate **8** and phosphate **7**, but 6-hydroxyl would be preferentially phosphorylated for its steric reasons as glucose phosphorylation (Fig. S25).

The yield of the phosphorylated sugars in the presence of borate **8** would be related to the structure of each sugar. Glucose **1** and galactose **11** showed high phosphorylation yields, based on the amount of residual sugar. When urea **9** combines with glucose **1** and galactose **11**, borate **8** retains these ureido-sugars in the chain form. The chain form of ureido-hexose combined with borate **8** has a hydroxyl group that can react with phosphate **7** (Fig. S26a,b). In contrast, mannose **10** and fructose **12** produced small amounts of phosphorylated products. In combination with borate **8**, ureido-mannose would become cyclic and lack a hydroxyl group that can react with phosphate **7** (Fig. S26c). Fructose **12** would combine with urea **9** at 2-carbonyl, forming 2-ureido-fructose. This ureido-fructose can isomerize into an unstable structure with ketone, even with borate, followed by decomposition, leading to the low yield of its phosphorylated molecule. (Fig. S27). Therefore, the yields of phosphorylated sugars would have been different depending on the structure of each sugar on the prebiotic Earth. This would have occurred even if the amount of each sugar supplied to the early Earth was almost the same regardless of the structure of each sugar.

**Figure 5:**
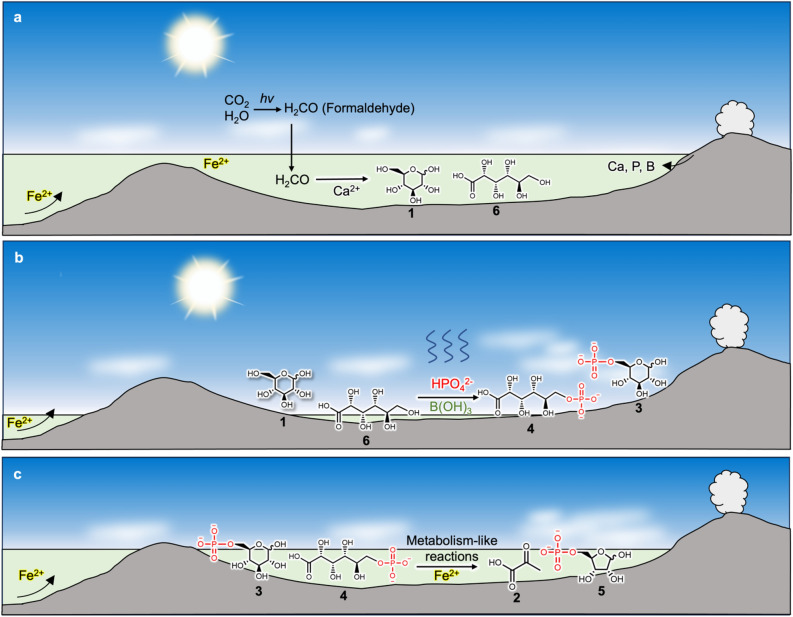
Proposed geological environment for the evolution of proto-glycolysis and proto-pentose phosphate pathway. (**a**) Formose-like reaction forms glucose 1 and gluconate 6. The starting molecule and catalyst (i.e., formaldehyde and Ca^2+^) were supplied from photochemical reaction and weathering of rocks, respectively. Borate 8, which was also supplied from the weathering or hydrothermal leaching of felsic rocks, contributes to the accumulation of polyoles in the formose-like reaction products. (**b**) Evaporation induced the phosphorylation of polyoles in the formose-like reaction products to form glucose 6-phosphate 3 and 6-phosphogluconate 4. Phosphate 7 was supplied from the weathering or hydrothermal leaching of felsic rocks, like Ca^2+^ and borate 8. Borate 8 also contributed to improving the yields and regioselectivity of the phosphorylation site. (**c**) Fe^2+^, which was available in the primordial ocean, promoted the metabolism-like reactions from glucose 6-phosphate 3 and 6-phosphogluconate 4 to form pyruvate 2 and ribose 5-phosphate 5.

The present results indicate that glucose **1** and gluconate **6** can be formed under the same conditions and that glucose **1** and gluconate **6** can be regioselectively phosphorylated to glucose 6-phosphate **3** and 6-phosphogluconate **4** exclusively in borate-rich evaporative environments (Fig. 5). Such evaporative basins would also have contributed to the accumulation of organic molecules and the promotion of dehydration reactions such as the phosphorylation and polymerization of amino acids^25,26,30,38,39^.

The selective formation of glucose 6-phosphate **3** and 6-phosphogluconate **4** by simple prebiotic reactions shown in this study consolidates non-enzymatic glycolysis-like reactions and pentose phosphate pathway-like reactions with pyruvate and ribose 5-phosphate **5** synthesis from these phosphates shown in previous research^4–6^. Previous studies have shown that the synthesis of pyruvate **2** and ribose 5-phosphate **5** is catalyzed by Fe^2+^^4–6^. Thus, these reactions can be assumed to have been initiated in the Fe^2+^-rich environment of early Earth. Fe^2+^ is presumed to have been a common cation in the ancient ocean before the oxidation of the primordial ocean by biological activities^43–46^. Furthermore, extraterrestrial delivery may have contributed to local iron enrichment^47^. In addition, iron-rich meteorite impacts have been proposed to form reduced organic molecules, including cyanide compounds, that can be a precursor of urea **9**^37,40,47^, which would have contributed to the phosphorylation and formation of other biological molecules^29,30,48,49^. Therefore, evaporative basins enriched in boron and iron would have been conducive for non-enzymatic glycolysis-like reactions and pentose phosphate pathway-like reactions on early Earth. These metabolism-like reactions can supply various biomolecules and their precursors, such as pyruvate **2** and ribose 5′-phosphate **5**. Pyruvate **2** is an intermediate of the TCA or rTCA cycle of modern life and can be a precursor of amino acids^11–13^. Ribose 5-phosphate **5** is a precursor of nucleotides, and previous studies have shown that nucleotides are formed via ribose 5-phosphate **5** under prebiotically plausible conditions^50^. Previous studies have discussed the possibility that some non-enzymatic metabolism-like reactions on the prebiotic Earth were inherited by the metabolism of ancient life^4–14^. Non-enzymatic glycolysis-like reactions and pentose phosphate pathway-like reactions consolidated by the present study support this hypothesis.

In conclusion, both glucose **1** and gluconate **6** were formed via formose-like reactions from formaldehyde and glycolaldehyde. These compounds were regioselectively phosphorylated at 6-hydroxyl to form glucose 6-phosphate **3** and 6-phosphogluconate **4** with borate **8** by thermal evaporation, suggesting that these compounds were formed in borate-rich evaporative environments on prebiotic Earth. This consolidates the progress of non-enzymatic glycolysis-like reactions and pentose phosphate pathway-like reactions, which can supply various biomolecules to the prebiotic Earth.

## Materials and methods

### Glucose and gluconic acid formation experiments

Glucose and gluconic acid formation were performed following a previously reported formose-like reaction method^19^. An aqueous solution (15 mL) containing 100 mM formaldehyde (Wako; Osaka, Japan), 10 mM glycolaldehyde (Sigma-Aldrich), and 10 mM Ca(OH)_2_ (Wako) was prepared in a polytetrafluoroethylene (PTFE) bottle. The solution was heated for 72 h at 95 °C under continuous stirring. An aliquot of the solution (100 µL) was dried using a rotary evaporator.

### Sugar derivatization procedure

Sugar derivatization was performed as previously described^19^. The dried samples were placed in 200 µL of pyridine solution (Wako) containing hydroxylammonium chloride (Wako) and heated at 90 °C for 1 h to convert the 1′ aldehyde of sugars into nitrile. Subsequently, 500 µL of acetic anhydrite (Wako) was added to the samples, followed by heating at 90 °C for 1 h to acetylate the hydroxyls. The samples were dried under N_2_ flow and added to 1 mL of dichloromethane (Wako). The dichloromethane fraction was washed with 1 mol/L HCl (Wako) and ultrapure water. Dichloromethane was replaced with a solvent containing hexane (Wako) and ethyl acetate (Wako).

### GC–MS analysis of formose-like reaction products

The derivatized sample was injected into a gas chromatography-mass spectrometer (Shimadzu GCMS-QP2010; Kyoto, Japan) equipped with a DB-17 ms column (60 m long, 025 µm thick, 0.25 mm ID; Agilent). The carrier gas was helium at a flow rate of 0.8 mL/min. The column oven temperature was initially 50 °C and kept for 2 min, ramped up to 120 °C at 15 °C/min and maintained for 5 min, followed by 160 °C at 4 °C/min and 170 °C at 3 °C/min. The inlet, interface, and ion source temperatures were set at 250, 250, and 200 °C, respectively. The yields were calculated based on their peak areas using calibration lines prepared with commercial standards (Fig. S28a).

### LC–MS/MS analysis of formose-like reaction products

For the analysis of sugar acids formed by a formose-like reaction, an aliquot of the sample was injected into an LC–MS/MS system (Shimadzu LCMS-8040) with a HILIC column (VT50-2D; Shodex) without any derivatization. The sample was eluted at 40 °C with an isocratic mode with 80% 25 mM ammonium formate and 20% acetonitrile with a total flow rate of 0.2 mL/min. Mass spectrometry was conducted in the negative ion mode with desolvation, source, and heat block temperatures of 250, 120, and 400 °C, respectively. The yields were calculated based on their peak areas using calibration lines prepared with commercial standards (Fig. S28b).

### Glucose and gluconic acid phosphorylation experiments

The phosphorylation experiments were conducted in Eppendorf tubes. The experimental conditions were similar to those previously reported for ribose phosphorylation^25,26^. An aqueous solution (20 µL) containing 20 mM glucose or gluconic acid (Wako), 160 mM Na_2_HPO_4_ (Wako), 40 mM boric acid (Wako), and 800 mM urea (Wako) was prepared. The solution was heated for 24 h at 80 or 95 °C with the lid kept open in an electric furnace. After the experiment, 200 µL of pure water and 4 µL of 95% sulfuric acid were added to separate borate from glucose phosphate or gluconic acid phosphate. For the glucose phosphorylation experiment, the sample added by acid was further heated at 90 °C for 1 h to separate urea from glucose phosphate, following a previously reported method^25,26^.

### LC–MS/MS analysis of phosphorylated products

An aliquot of the sample was injected into an LC–MS/MS system (Shimadzu LCMS-8040) equipped with a HILIC column (VT50-2D; Shodex). The glucose phosphorylation products and ureido-glucose phosphate were eluted at 60 °C in the isocratic mode with 80% 25 mM ammonium formate and 20% acetonitrile at a total flow rate of 0.2 mL/min. To analyze gluconate phosphorylation products, 25 mM ammonium formate buffered at pH 3 by formic acid was employed instead of its unbuffered solution. Mass spectrometry was conducted in the negative ion mode with desolvation, source, and heat block temperatures of 250, 120, and 400 °C, respectively. The yields were calculated based on their peak areas using calibration lines prepared with commercial standards (Fig. S29).

### LC–MS analysis of residual sugars and sugar acids

An aliquot of the sample was injected into an LC–MS/MS system (Shimadzu LCMS-8040) equipped with a HILIC column (VT50-2D; Shodex). For residual sugar analysis, the samples were eluted at 45 °C in the isocratic mode with 30% 25 mM ammonium formate and 70% pure acetonitrile at a total flow rate of 0.2 mL/min. For residual sugar acid analysis, the samples were eluted at 60 °C in the isocratic mode with 80% 25 mM ammonium formate buffered at pH 3 by formic acid and 20% acetonitrile at a total flow rate of 0.2 mL/min. Mass spectrometry was conducted in the negative ion mode with desolvation, source, and heat block temperatures of 250, 120, and 400 °C, respectively. The yields were calculated based on their peak areas using calibration lines prepared with commercial standards (Figs. S30 and S31).

### ^31^P-NMR analysis of glucose phosphate and gluconic acid phosphate

^31^P-NMR chemical shifts were acquired using a Bruker AVANCE III 500 spectrometer. We performed the following experiment to obtain a highly concentrated sample for the detection of ^31^P-NMR chemical shifts. The 20 µL aqueous solution containing 200 mM glucose or gluconic acid, 400 mM boric acid, 1.6 M dipotassium phosphate, and 4 M urea was heated and dried for 24 h at 80 °C. After the experiments, the sample with glucose was added to a sulfuric acid solution and heated at 90 °C for 1 h. Before NMR measurement, NaOH solution was added to the sample to adjust the pH to neutral. The sample containing gluconic acid was dissolved in pure water, followed by ^31^P-NMR analysis without any pre-analysis process.

### ^1^H-NMR and ^13^C-NMR analysis of ureido-glucose and ureido-glucose phosphate

^1^H-NMR and ^13^C-NMR chemical shifts were acquired using a JEOL JNM-ECZL 700G spectrometer. We performed the following experiment to obtain a highly concentrated ureido-glucose to detect ^13^C-NMR chemical shifts. The 100 µL aqueous solution containing 200 mM glucose, 400 mM boric acid, and 800 mM urea was heated and dried for 24 h at 80 °C. The dried residue was dissolved in D_2_O, followed by NMR analyses. Ureido-glucose phosphate was obtained in the same manner as the reaction for ^31^P-NMR described above. The dried residue was dissolved in D_2_O, followed by NMR analysis without acid hydrolysis.

### Direct infusion mass spectrometry analysis of ureido-glucose combined with borate

Direct infusion mass spectrometry was performed using an MS system (Shimadzu LCMS-8040) combined with a Harvard syringe pump (PUMP 11 ELITE). The following experiment was performed to obtain ureido-glucose combined with borates. The 100 µL aqueous solution containing 100 mM glucose, 100 mM urea, and 800 mM boric acid was heated and dried for 12 h at 80 °C. The dried residue was dissolved in 1000 µL ultrapure water, followed by the direct infusion MS analyses. The flow rate of the syringe pump was constantly 0.05 mL/min, flowing into the MS system coupled with 0.05 mL/min pure acetonitrile coming from the LC system of LCMS-8040. Mass spectrometry was performed in the ESI negative mode with desolvation, source, and heat block temperatures set at 250, 120, and 400 °C, respectively.

### Supplementary Information

Supplementary Figures.

## Data Availability

All data are available in the main text or supplementary materials.
