# Supplementary material for: Hexose phosphorylation for a non-enzymatic glycolysis and pentose phosphate pathway on early Earth
Source: Sci Rep. 2024 Jan 2;14:264. doi: 10.1038/s41598-023-50743-8 (PMC10762079; doi:10.1038/s41598-023-50743-8)
Supplement: Supplementary file 1 — Supplementary Figures. [file 41598_2023_50743_MOESM1_ESM.pdf]

Supplementary Information for

# Hexose phosphorylation for a non-enzymatic glycolysis and pentose phosphate pathway on early Earth

Yuta Hirakawa*, Takeshi Kakegawa, Yoshihiro Furukawa

*Corresponding author. Email: yuta.hirakawa.s2@dc.tohoku.ac.jp

**This PDF file includes:**

Figs. S1 to S31

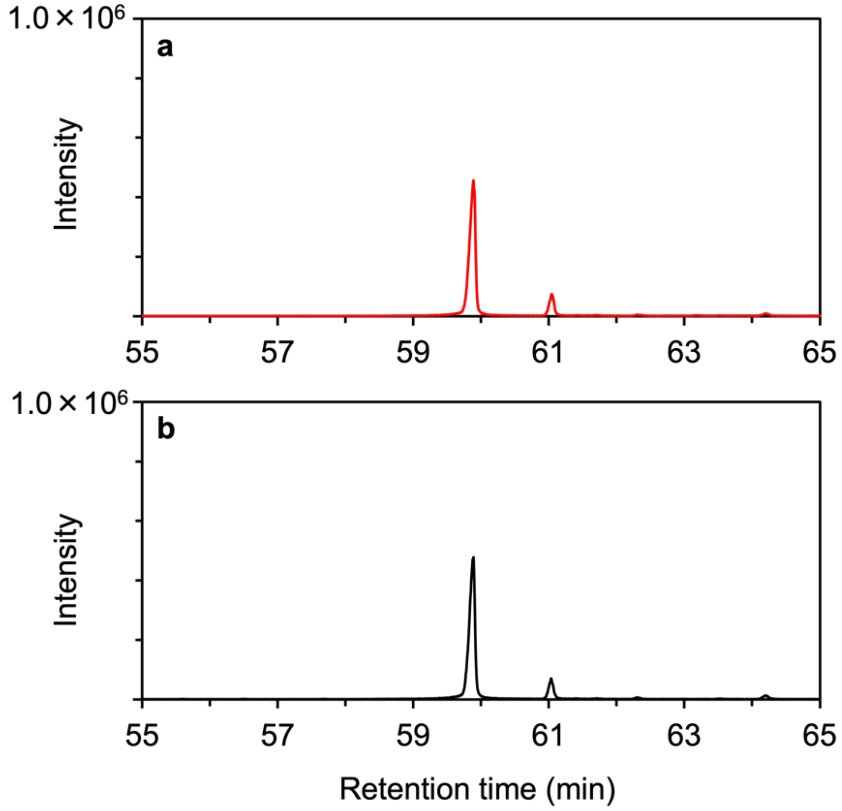


**Fig. S1. Identification of fructose in formose-like reaction products by GC-MS. (a)** GC-MS chromatogram of a derivatized formose reaction product (m/z: 110). **(b)** GC-MS chromatogram of derivatized standard fructose (m/z: 110).

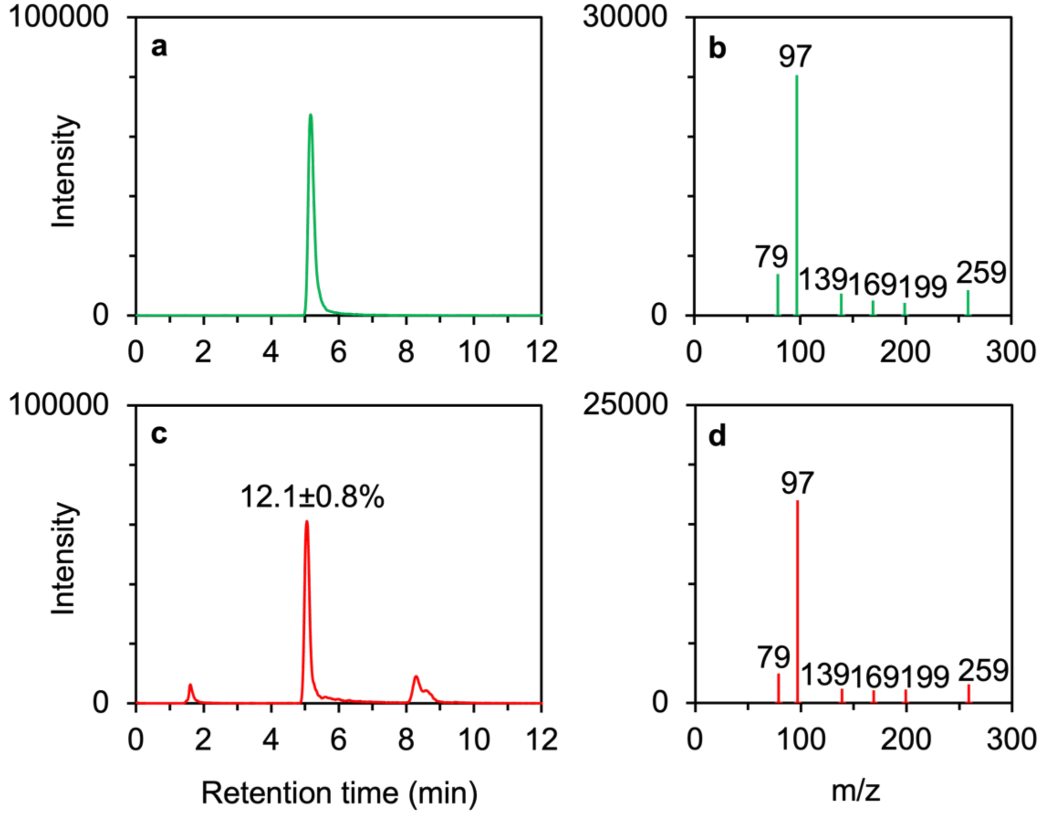


**Fig. S2. LC-MS/MS analysis of glucose phosphate in the phosphorylation experiment at 95 °C (m/z: 259>97). (a)** MRM chromatogram of the standard of glucose 6-phosphate **3**. **(b)** Fragment pattern spectrum of the glucose 6-phosphate **3**. **(c)** MRM chromatogram of the experimental product at 95 °C. **(d)** Fragment pattern spectrum of the experimental product at 95 °C.

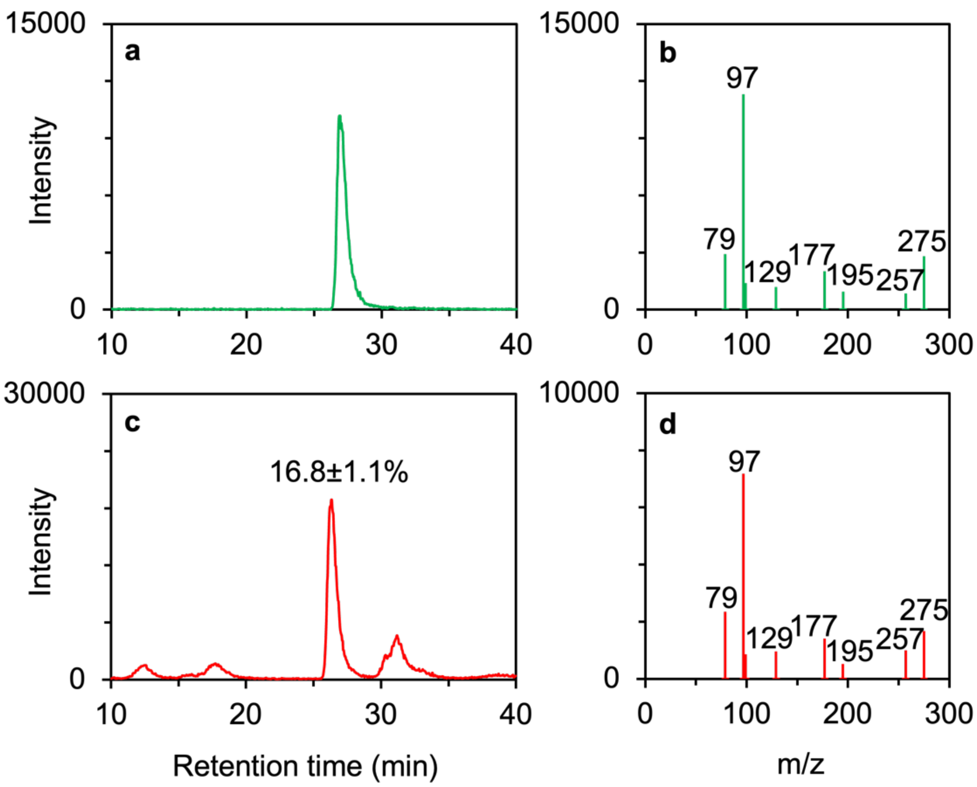


**Fig. S3. LC-MS/MS analysis of phosphogluconate in the phosphorylation experiment at 95 °C (m/z: 275>97). (a)** MRM chromatogram of the standard of 6-phosphogluconate **4**. **(b)** Fragment pattern spectrum of the 6-phosphogluconate **4**. **(c)** MRM chromatogram of the experimental product at 95 °C. **(d)** Fragment pattern spectrum of the experimental product at 95 °C.

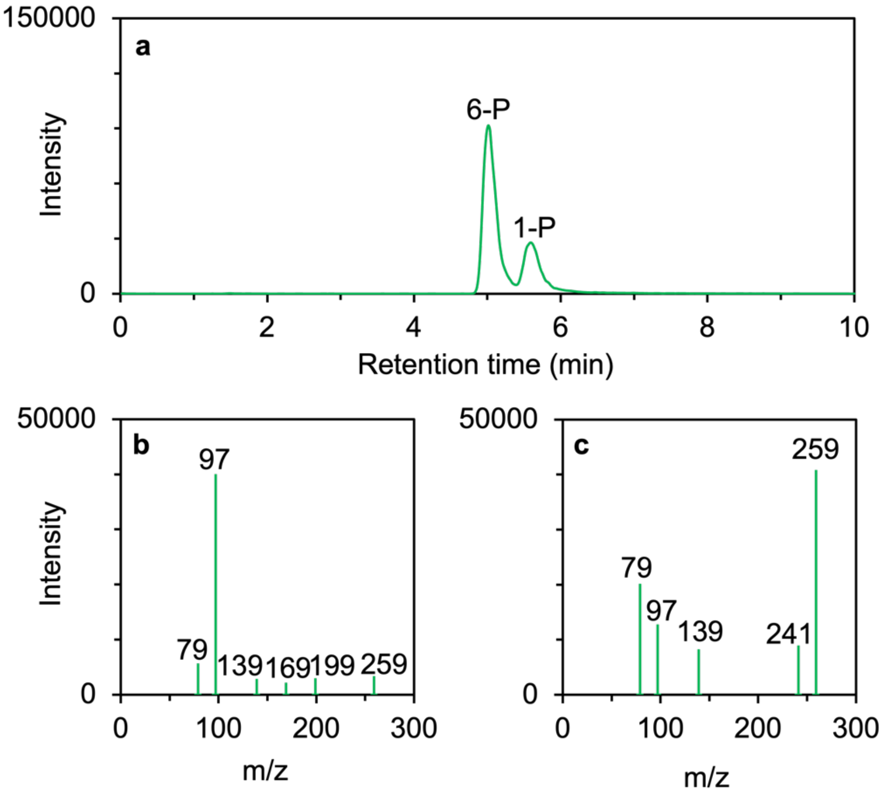


**Fig. S4. LC-MS/MS analysis of the standard of glucose phosphates (m/z: 259>97). (a)** MRM chromatogram of the standard of glucose 6-phosphate **3** (6-P) and glucose 1-phosphate (1-P). **(b)** Fragment pattern spectrum of the standard of glucose 6-phosphate **3**. **(c)** Fragment pattern spectrum of the standard of glucose 1-phosphate.

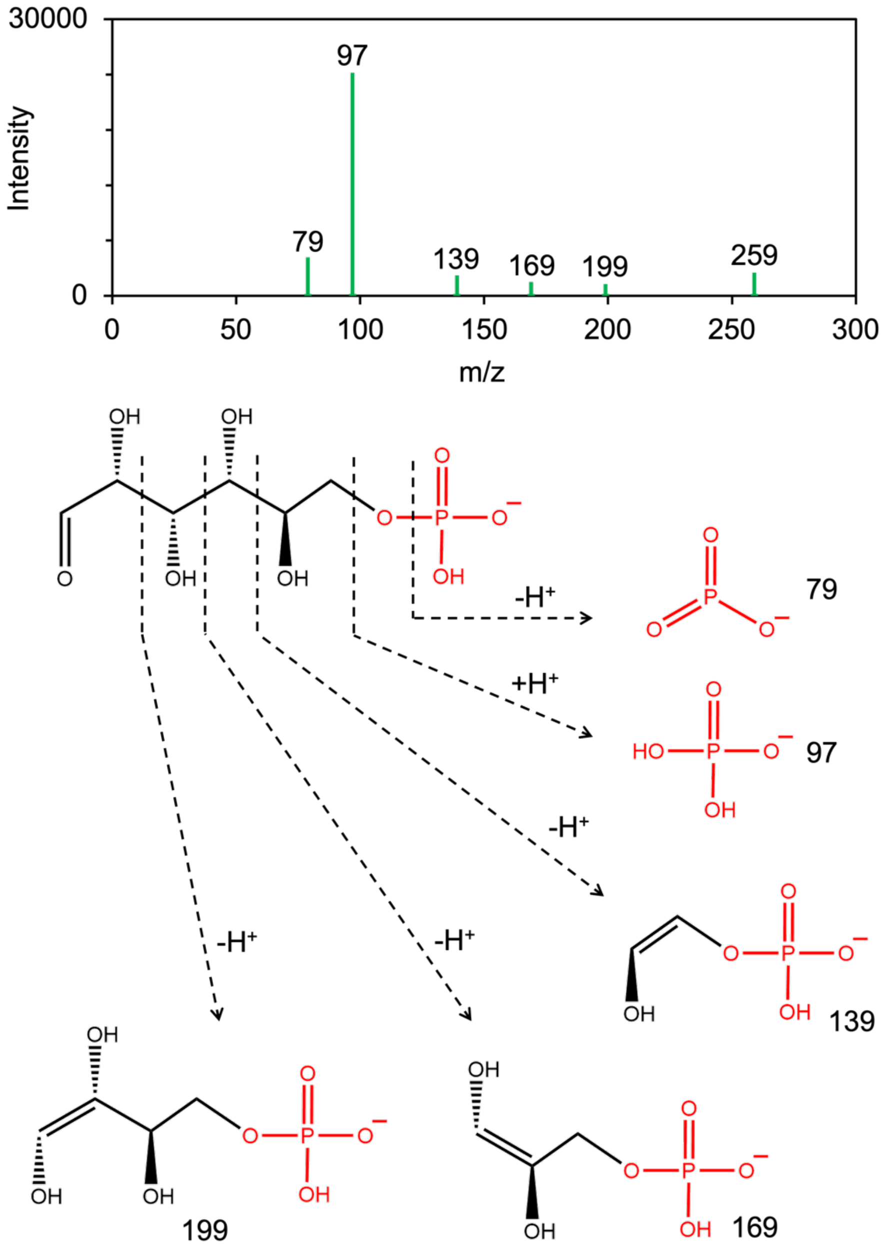


**Fig. S5. Fragmentation pattern of glucose 6-phosphate.** The molecular ion mass is 259. Fragments from phosphate moieties (79 and 97) show intense signals. The signals of 199, 169, and 139 are created by the fragmentation in glucose. The signal 139 is characteristic of the 6-phosphate and 5-phosphate. Phosphates combined at 2, 3, or 4-hydroxyl do not form the fragment 139.

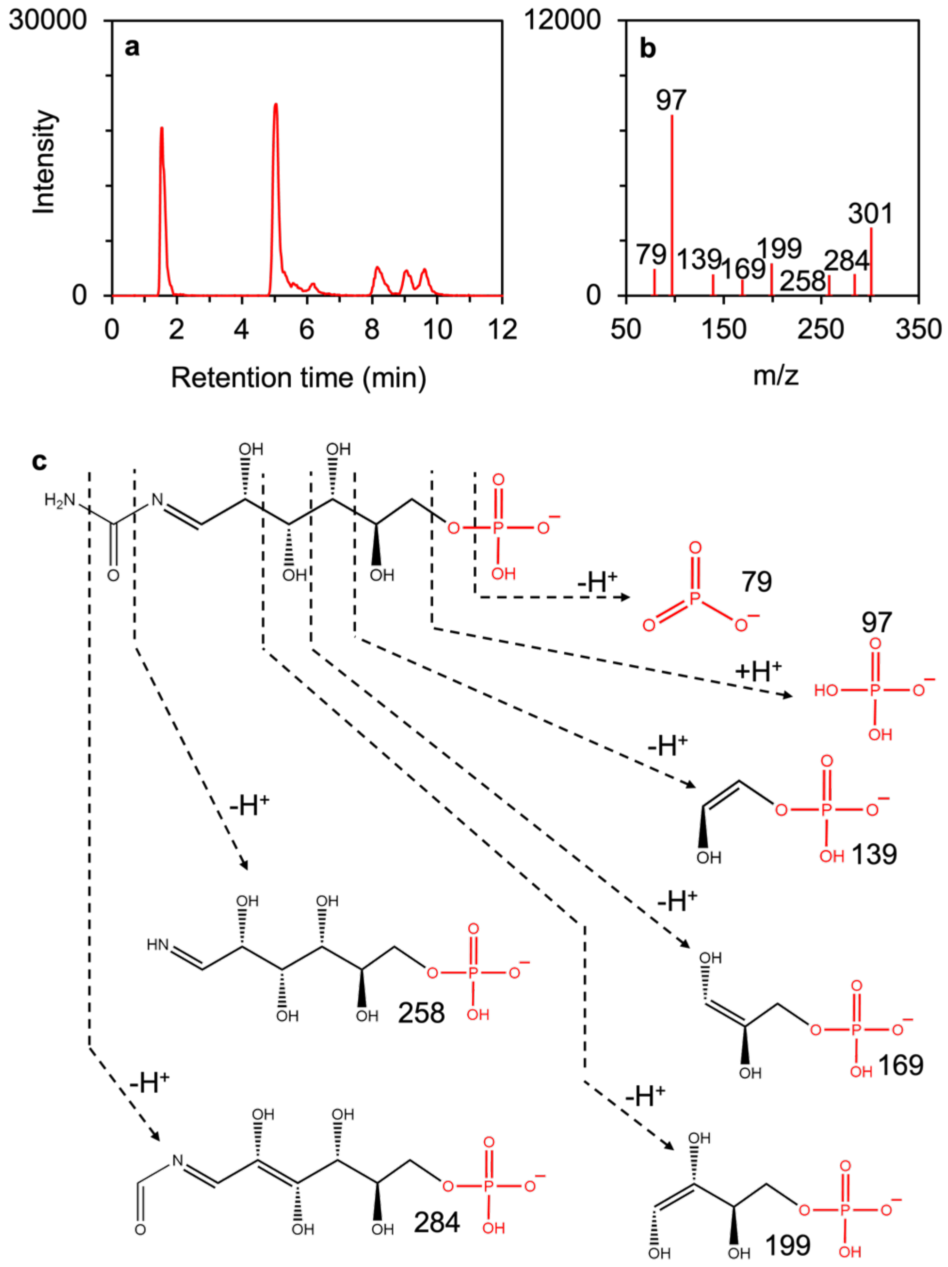


**Fig. S6. LC-MS/MS analysis of ureido-glucose phosphate (m/z: 301>97). (a)** MRM chromatogram of phosphorylation products without acid hydrolysis. **(b)** Fragment pattern spectrum of the peak around 5 min in (a).

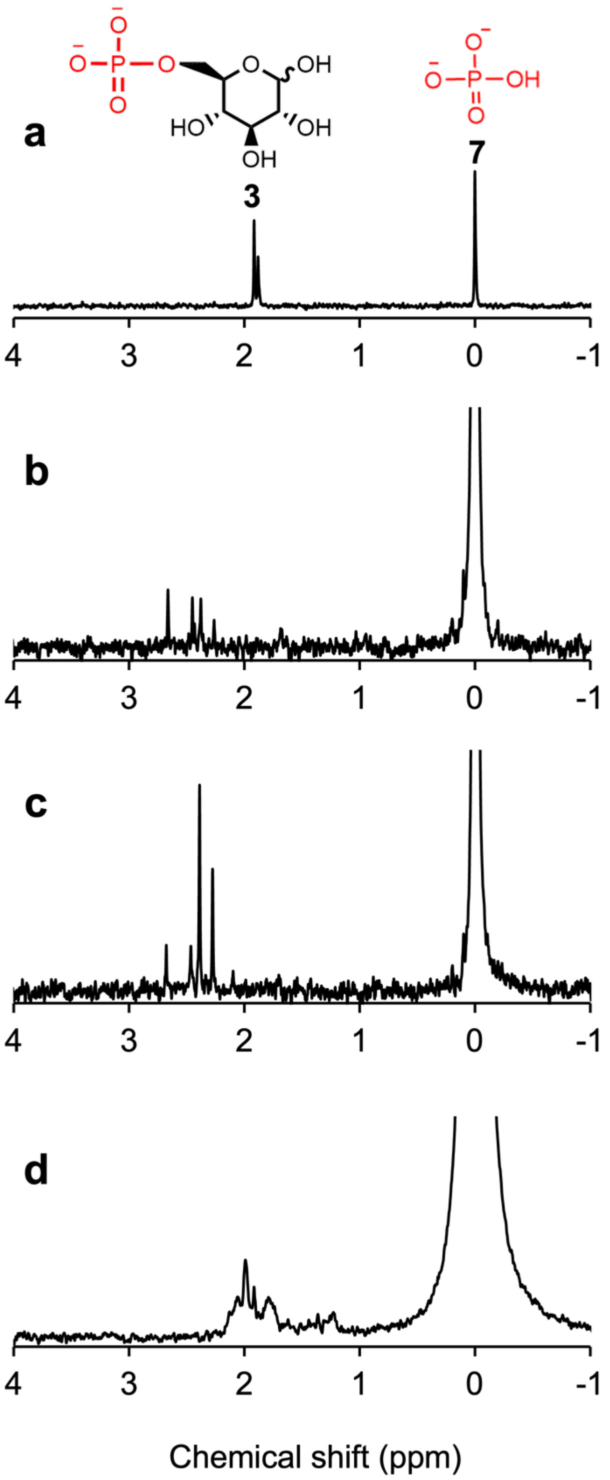


**Fig. S7. $^{31}$P-NMR spectra of glucose 6-phosphate.** (**a**) Standard phosphates. (**b**) Reaction products. (**c**) Reaction products with the added standard phosphates. **(d)** Reaction product without acid hydrolysis.

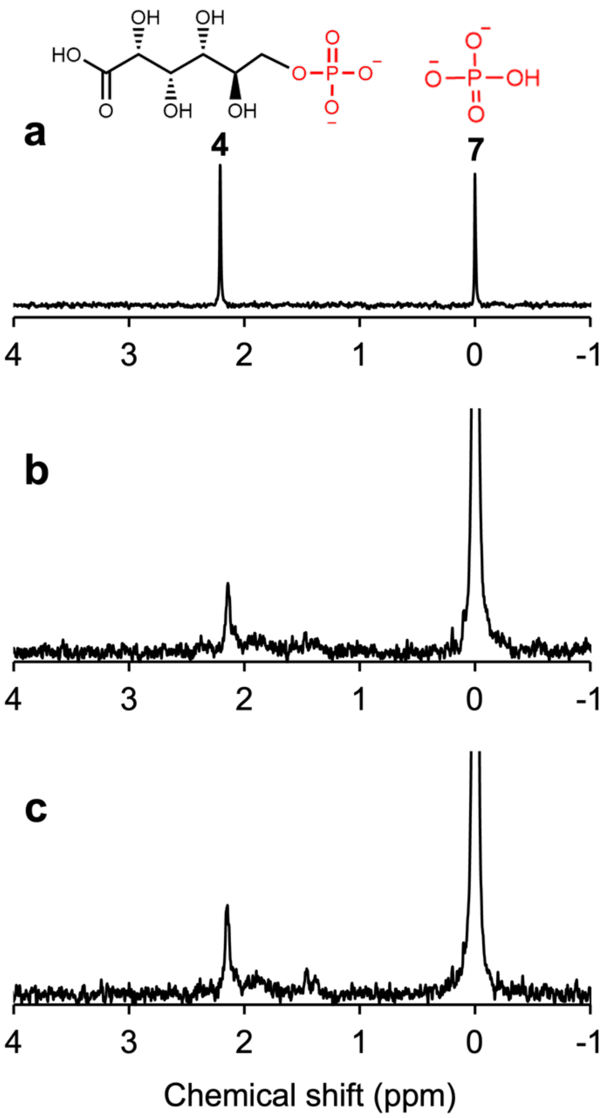


**Fig. S8. $^{31}$P-NMR spectra of phosphogluconate.** (**a**) Standard phosphates. (**b**) Reaction products. (**c**) Reaction products with the added standard phosphates.

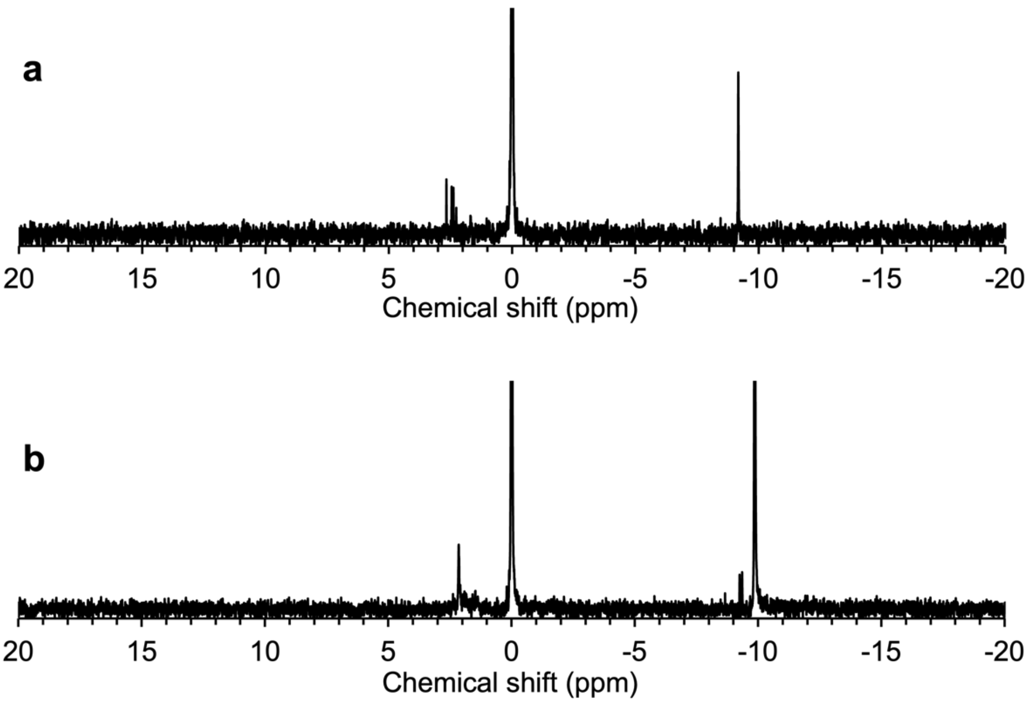


**Fig. S9. Full $^{31}$P-NMR spectra of phosphorylation products. (a)** glucose phosphate. **(b)** phosphogluconate.

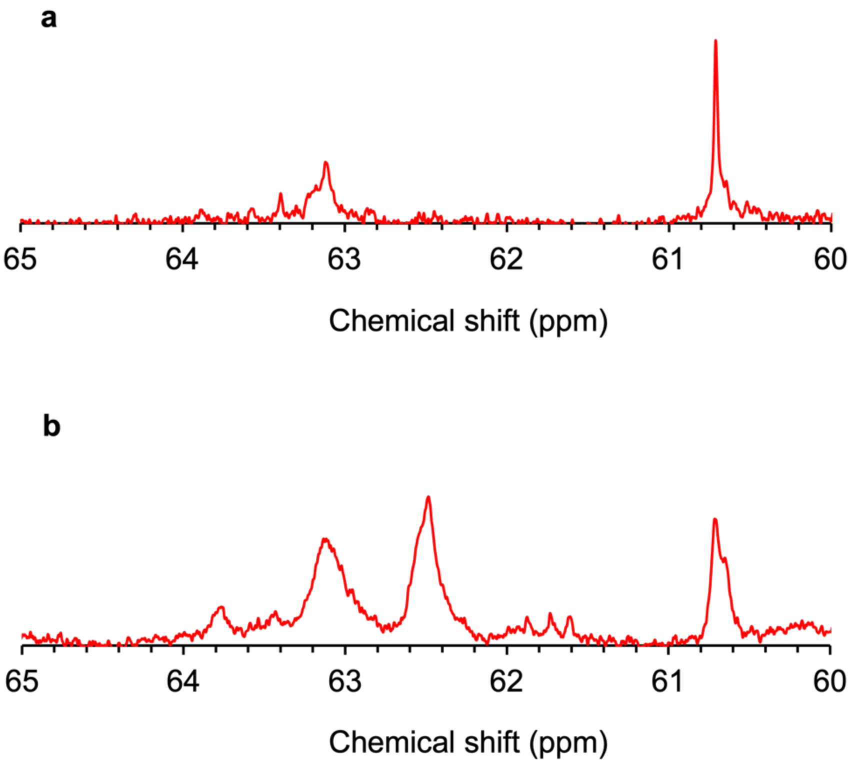


**Fig. S10. Comparison of $^{13}$C-NMR spectra of product ureido-glucose and ureido-glucose phosphate around 6-carbon. (a)** Spectrum of product ureido-glucose. **(b)** Spectrum of product ureido-glucose phosphate with remaining ureido-glucose.

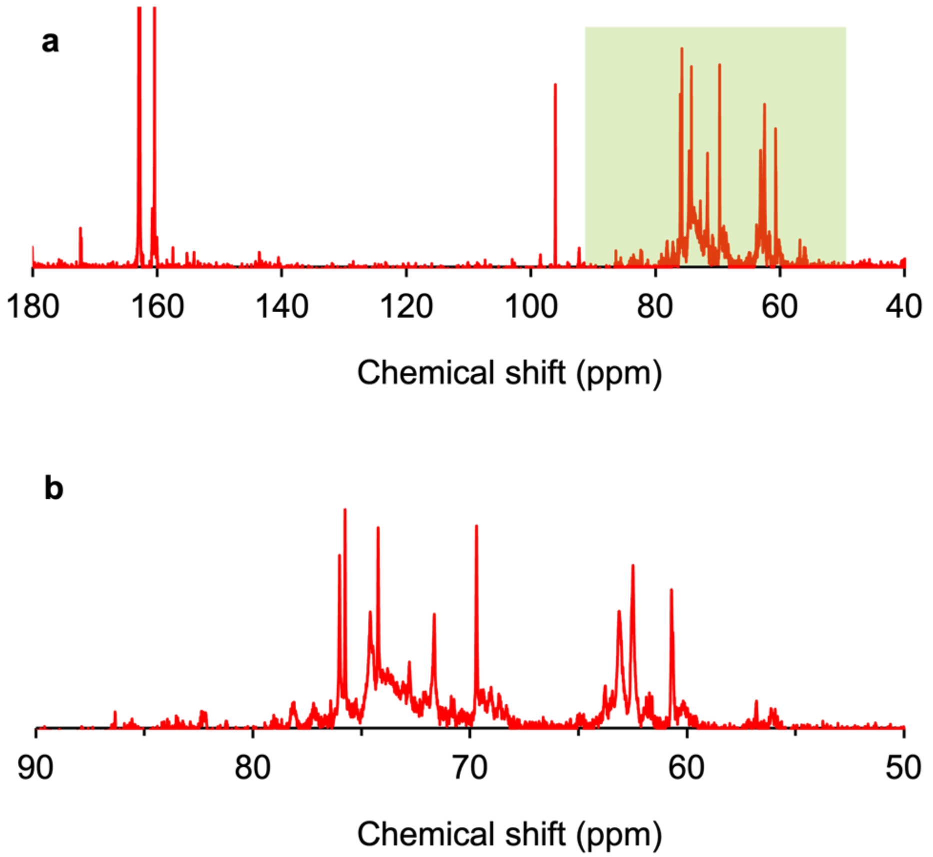


**Fig. S11. $^{13}$C-NMR spectra of product ureido-glucose phosphate. (a)** Full spectrum of the experimental product. **(b)** Enlarged spectrum view of chemical shifts from glucose of the experimental product (green-shaded area in **a**).

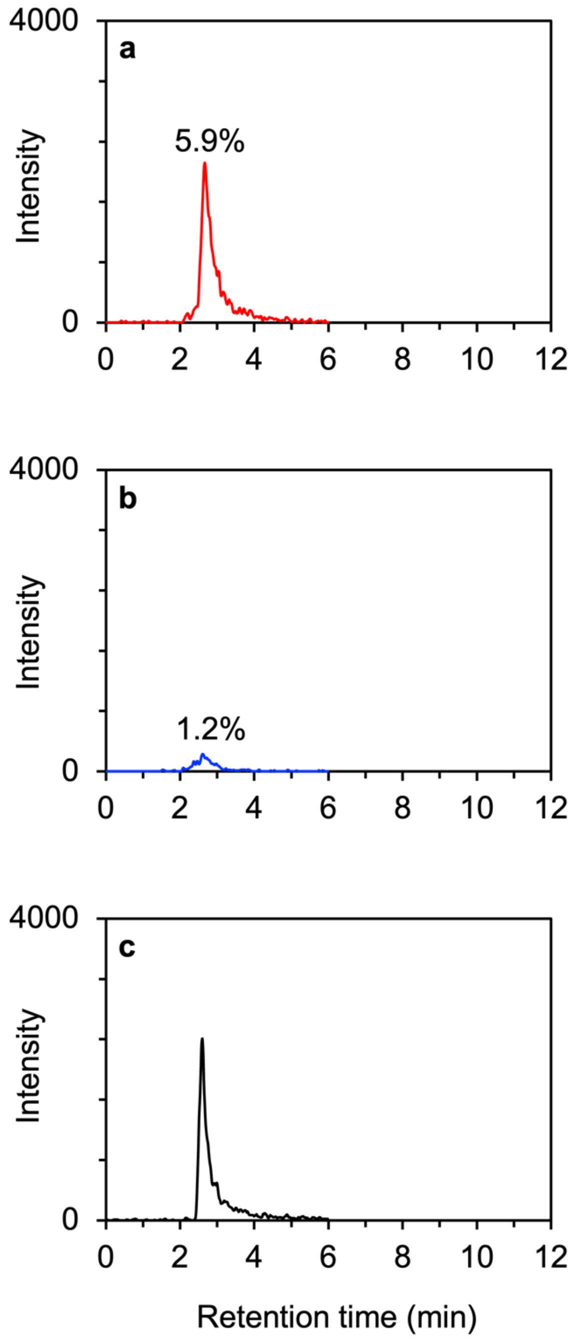


**Fig. S12. LC-MS/MS analysis of residual glucose (m/z: 179>59). (a)** Residual glucose in the experiment with borate. The yields represent the average of triplicate experiments. **(b)** Residual glucose in the experiment without borate. The yields represent the average of triplicate experiments. **(c)** The standard of glucose.

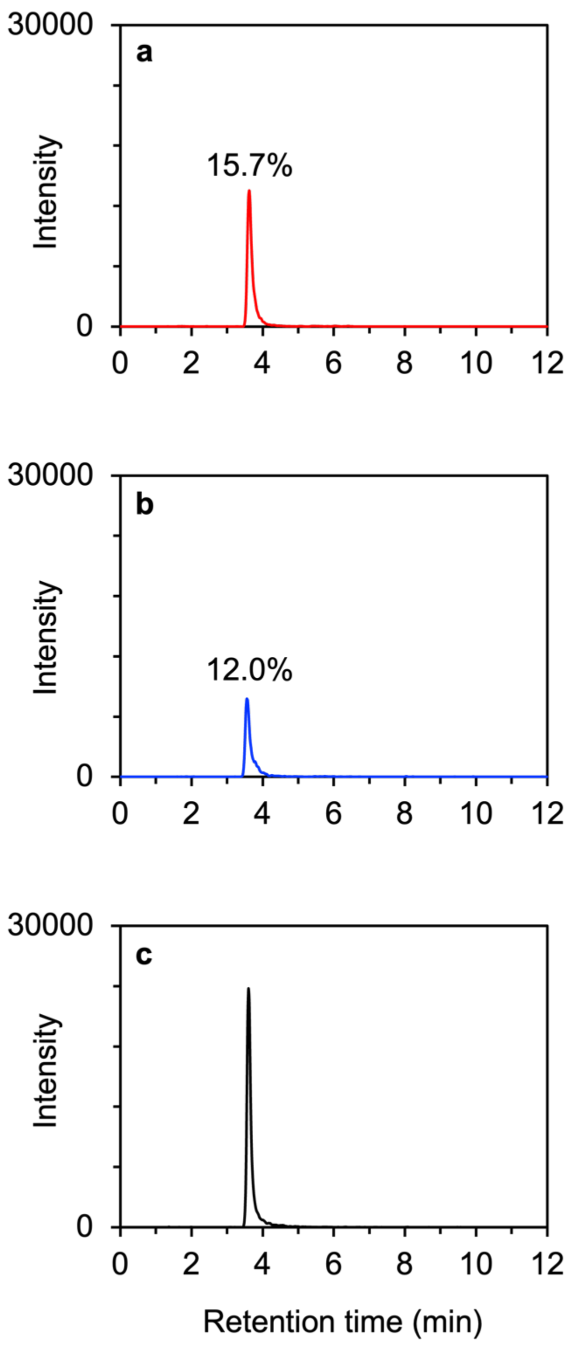


**Fig. S13. LC-MS/MS analysis of residual gluconic acid in the phosphorylation experiments (m/z: 195>75). (a)** Residual gluconic acid in the experiment with borate. The yields represent the average of triplicate experiments. **(b)** Residual gluconic acid in the experiment without borate. The yields represent the average of triplicate experiments. **(c)** The standard of gluconic acid.

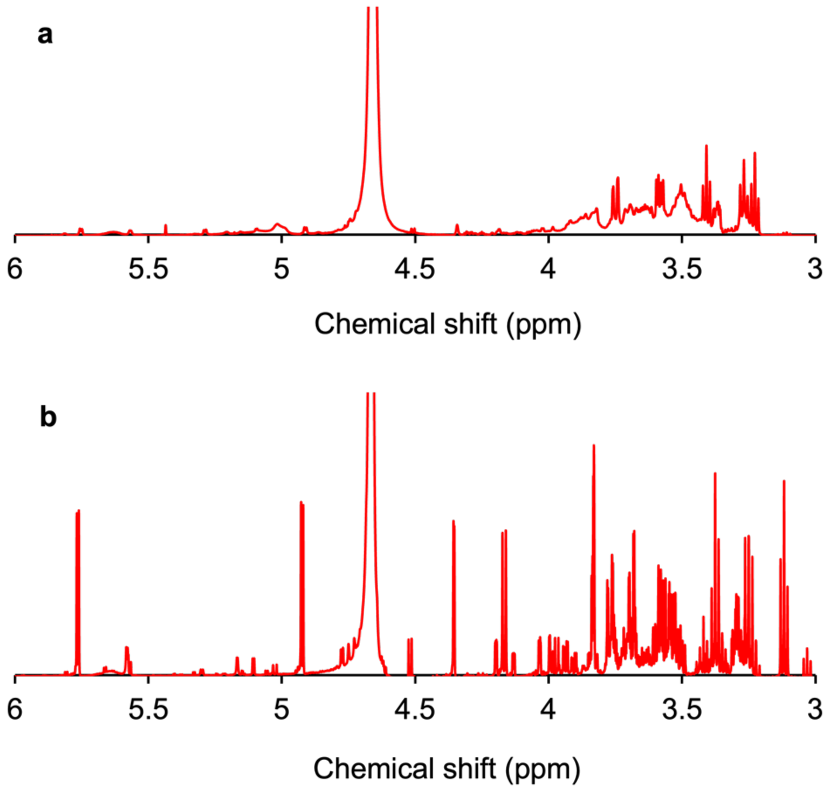


**Fig. S14. $^1$H-NMR spectra of the condensation products of glucose and urea. (a)** Experimental product in the presence of borate. **(b)** Experimental product in the absence of borate.

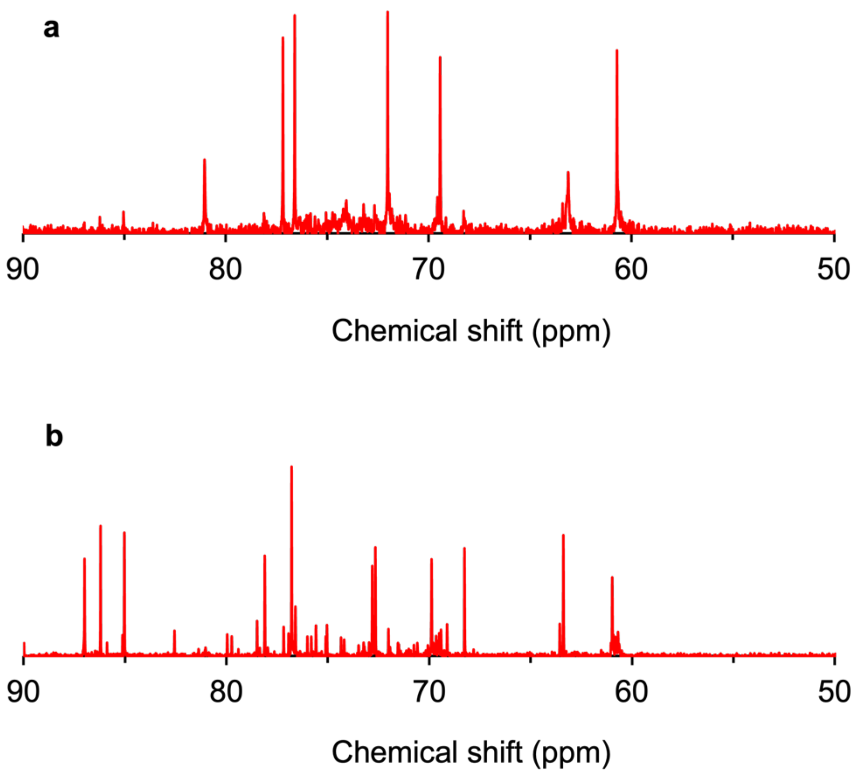


**Fig. S15. $^{13}C$-NMR of the condensation products of glucose and urea. (a)** Experimental product in the presence of borate. **(b)** Experimental product in the absence of borate.

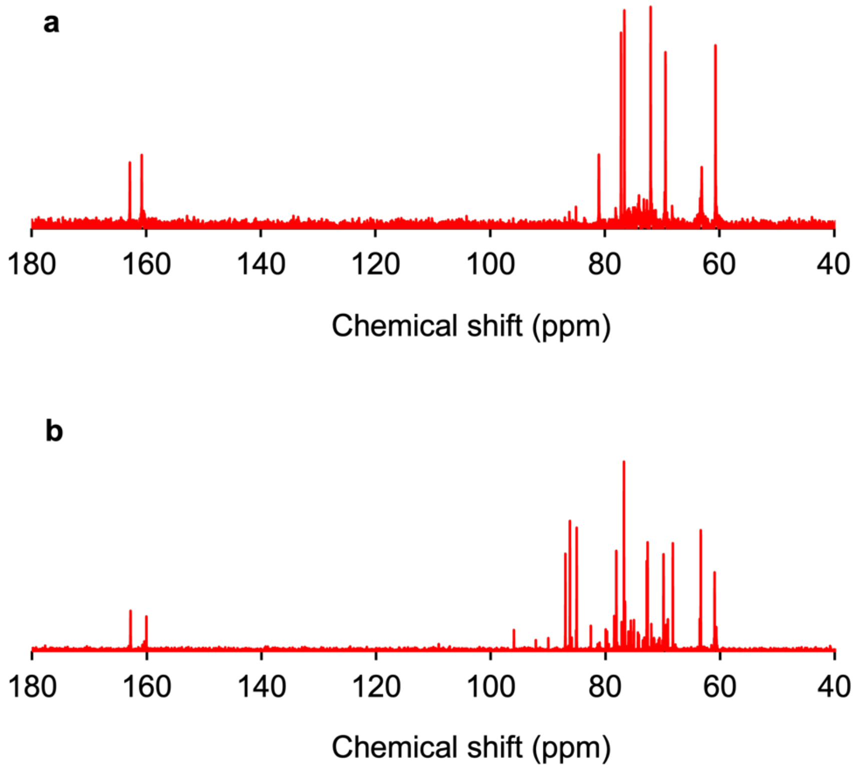


**Fig. S16. Full $^{13}C$-NMR spectra of condensation products of glucose and urea. (a)** Experimental product in the presence of borate. **(b)** Experimental product in the absence of borate.

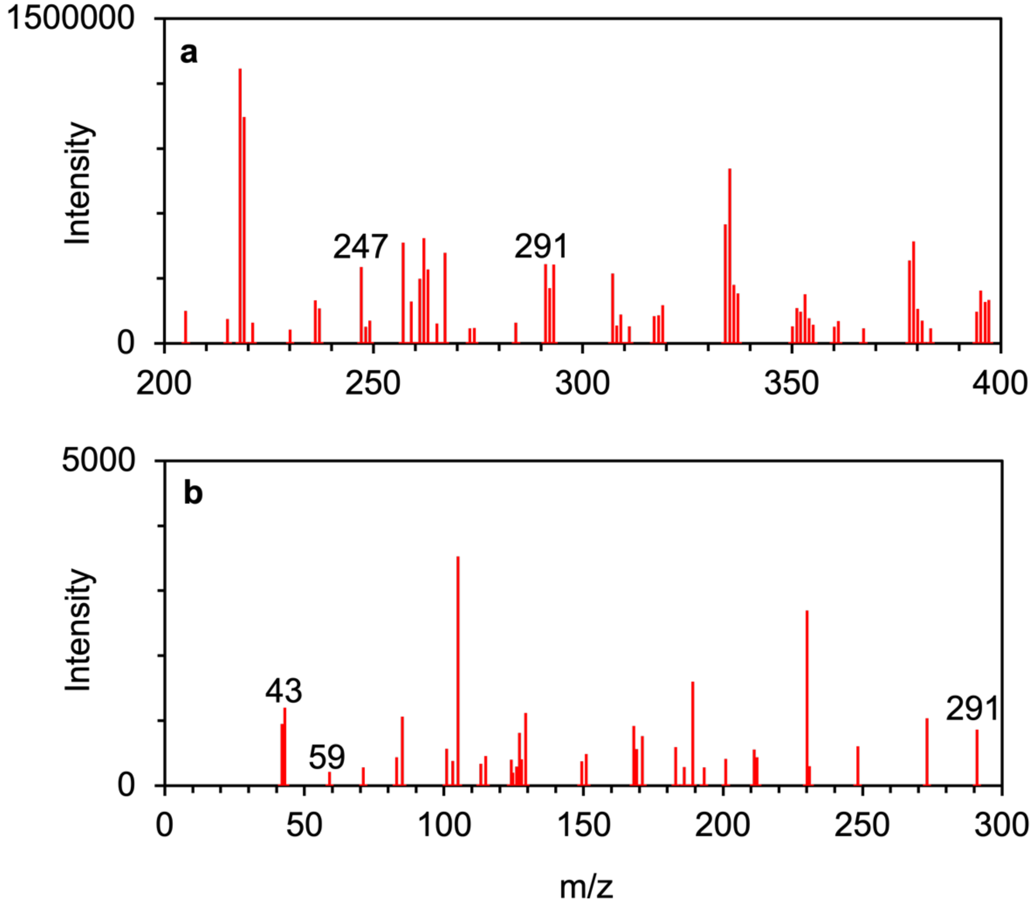


**Fig. S17. MS spectra of the direct infusion analysis of glucose and urea condensation products in the presence of borate. (a)** Negative ESI-MS spectrum showing the formation of ureido-glucose combined with one or two borates. The m/z signal at 243 Da corresponds to ureido-glucose combining one boric acid. The m/z signal at 291 Da corresponds to ureido-glucose combining one boric acid and one borate. Other major peaks are attributed to polyborates. **(b)** The fragmentation spectrum of the precursor ion 291 Da that corresponds to the mass of ureido-glucose combined with two borate molecules. The m/z signals of 43 and 59 are attributed to fragments from urea.

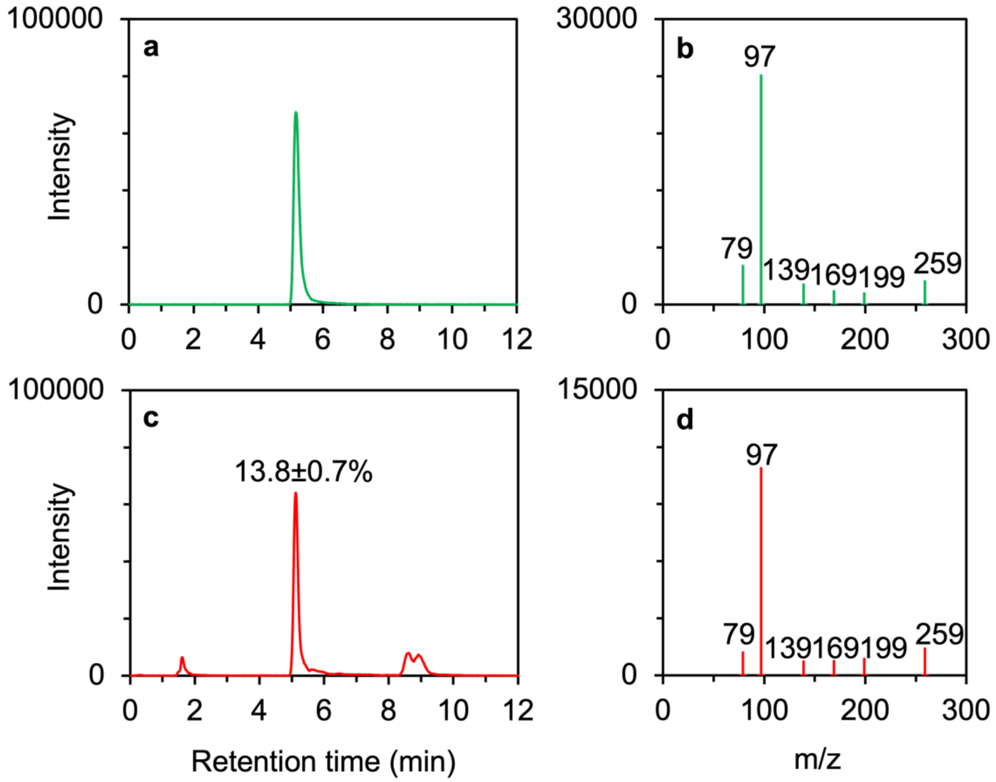


**Fig. S18. LC-MS/MS analysis of glucose phosphate in the phosphorylation experiment in the presence of glucose, gluconate, and $Ca^{2+}$. (a)** MRM chromatogram of the standard of glucose 6-phosphate **3** (m/z: 259>97). **(b)** Fragmentation spectrum of the glucose 6-phosphate **3**. **(c)** MRM chromatogram of the experimental product (m/z: 259>97). **(d)** Fragmentation spectrum of the experimental product.

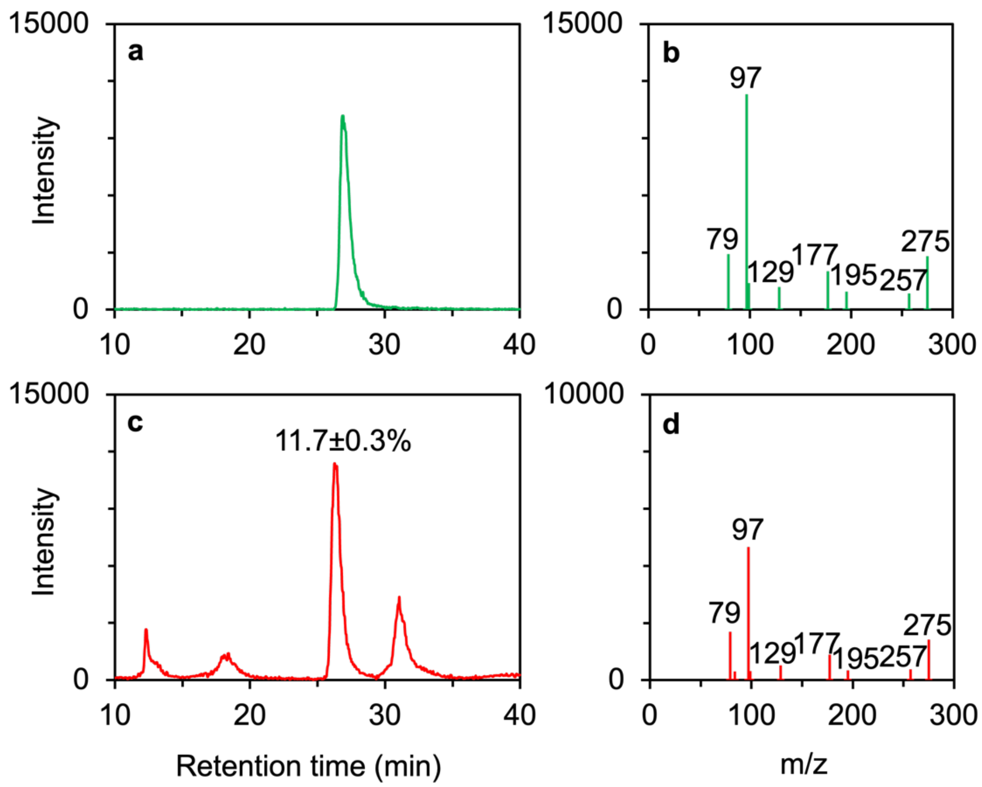


**Fig. S19 LC-MS/MS analysis of phosphogluconate in the phosphorylation experiment in the presence of glucose, gluconate, and $Ca^{2+}$. (a)** MRM chromatogram of the standard of 6-phosphogluconate **4** (m/z: 275>97). **(b)** Fragment pattern spectrum of the 6-phosphogluconate **4**. **(c)** MRM chromatogram of the experimental product (m/z: 275>97). **(d)** Fragment pattern spectrum of the experimental product.

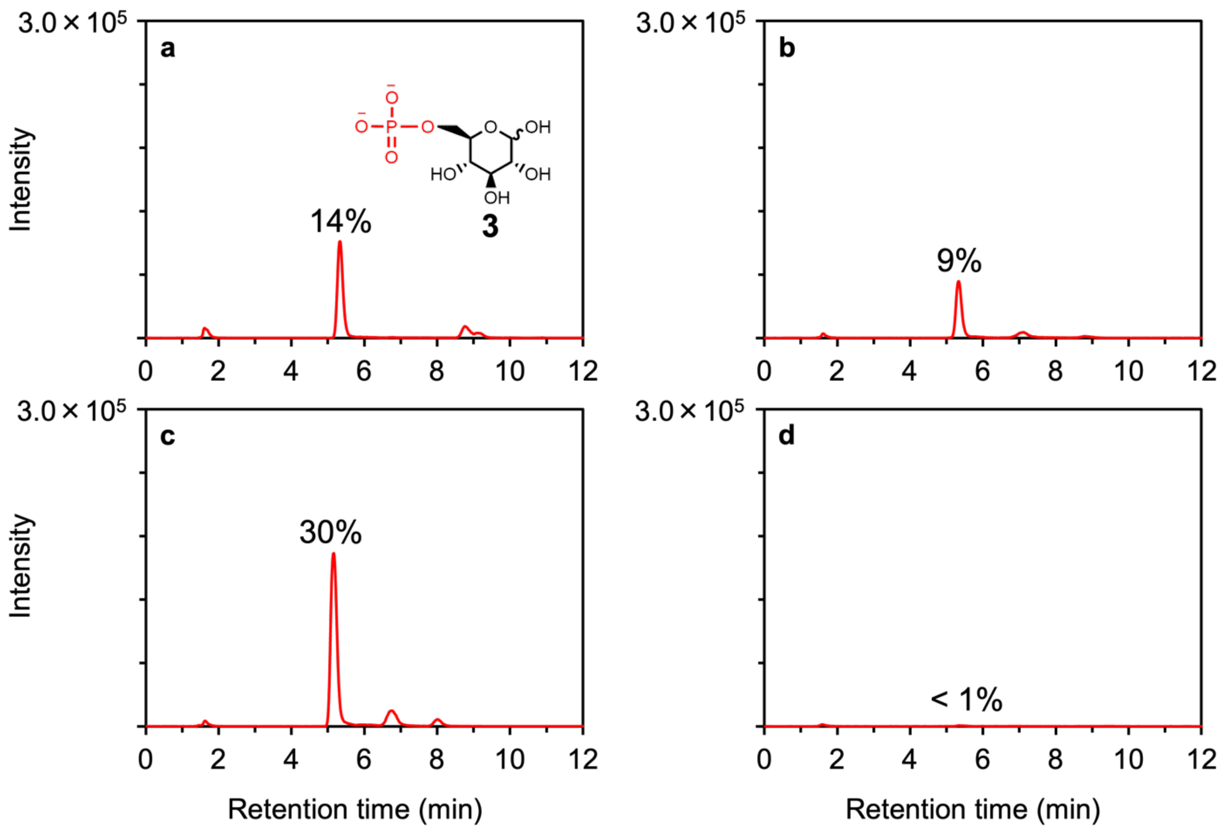


**Fig. S20. Yields of hexose phosphates in the phosphorylation experiment with borate (m/z: 259>97). (a)** Glucose phosphate. **(b)** Mannose phosphate. **(c)** Galactose phosphate. **(d)** Fructose phosphate. The yields represent the average of triplicated experiments.

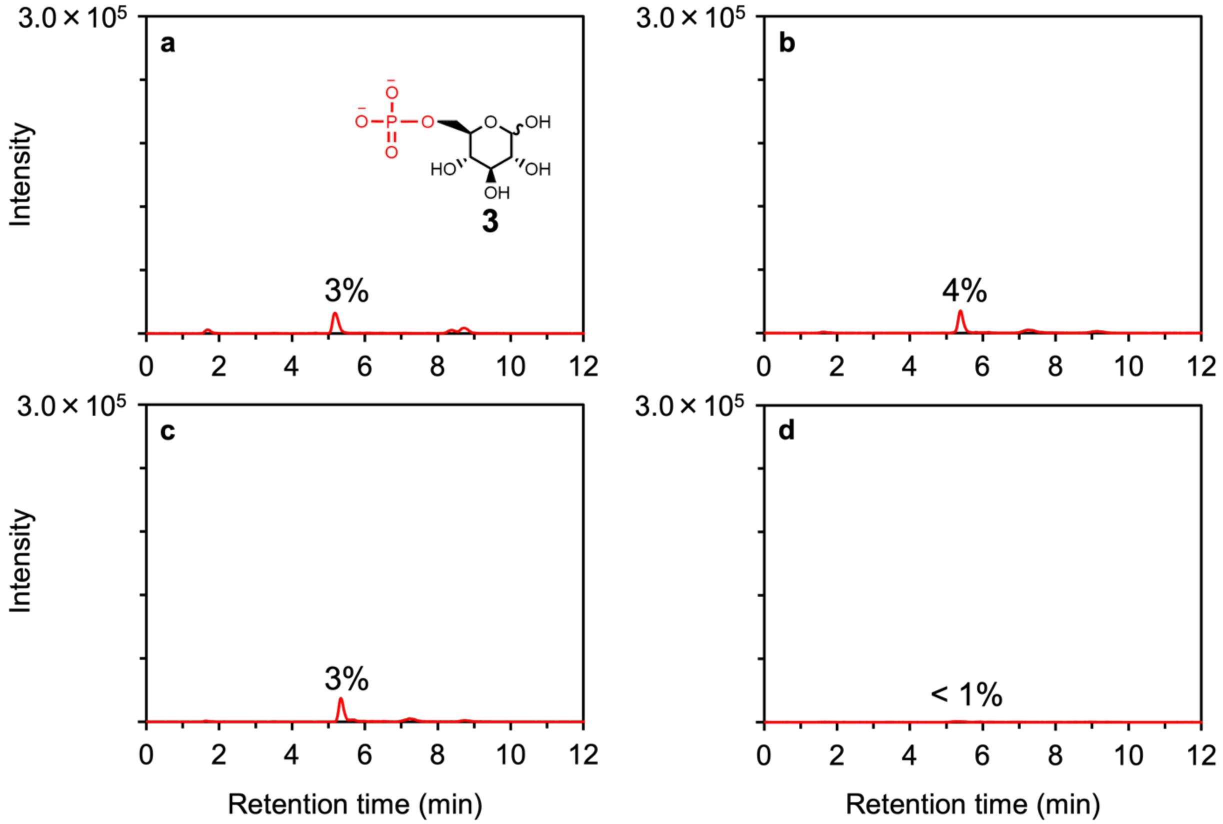


**Fig. S21. Yields of hexose phosphates in the phosphorylation experiment without borate (m/z: 259>97). (a)** Glucose phosphate **(b)** Mannose phosphate. **(c)** Galactose phosphate. **(d)** Fructose phosphate. The yields represent the average of triplicated experiments.

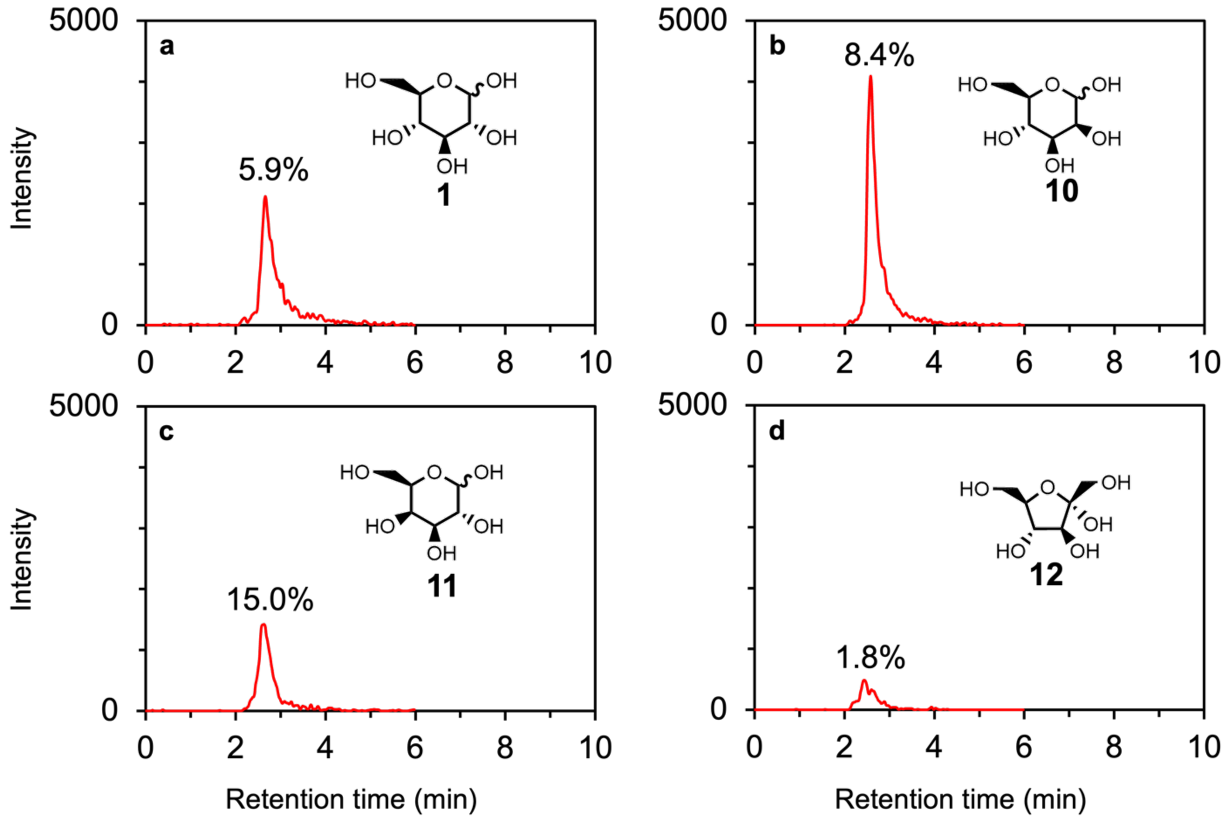


**Fig. S22. Residual amounts of hexoses after the phosphorylation experiment with borate (m/z: 179>59). (a)** Residual glucose. **(b)** Residual mannose. **(c)** Residual galactose. **(d)** Residual fructose. The yields represent the average of triplicated experiments.

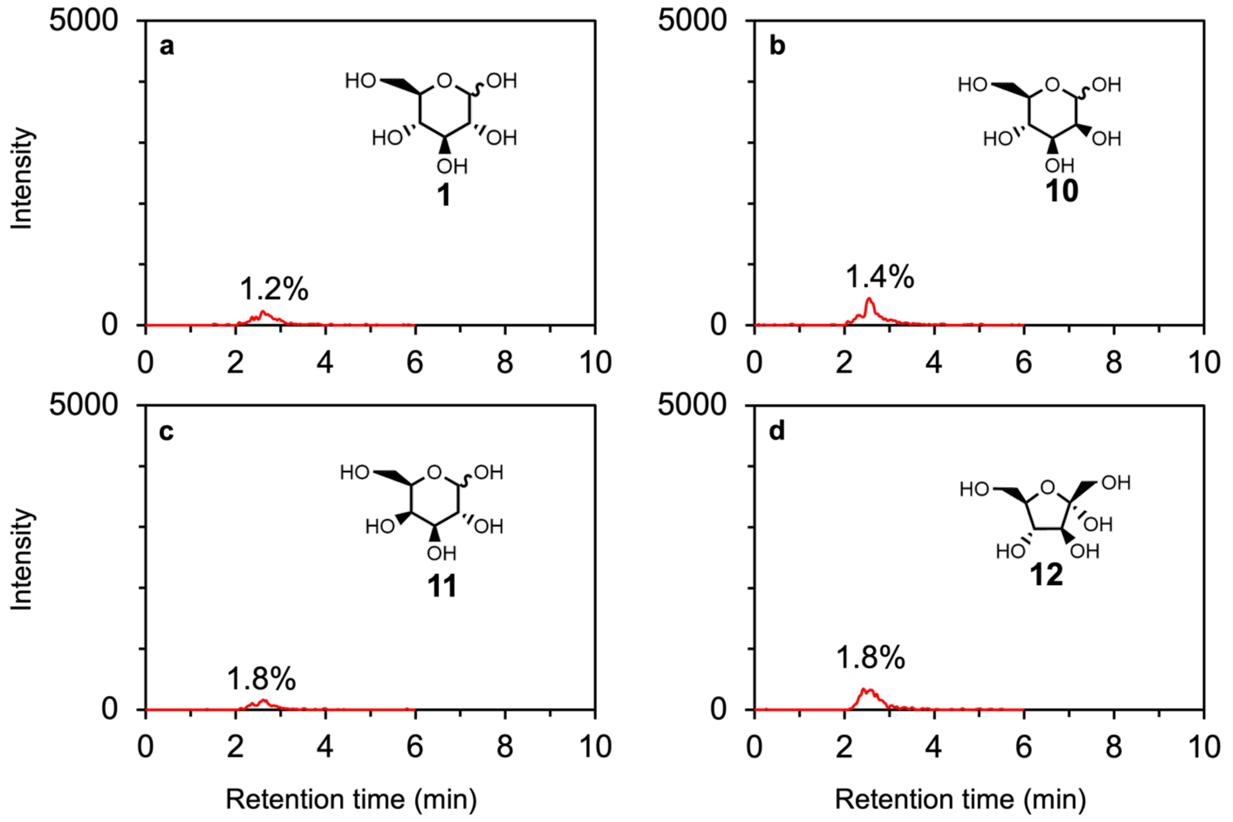


**Fig. S23. Residual amounts of hexoses after the phosphorylation experiment without borate (m/z: 179>59). (a)** Residual glucose. **(b)** Residual mannose. **(c)** Residual galactose. **(d)** Residual fructose. The yields represent the average of triplicated experiments.

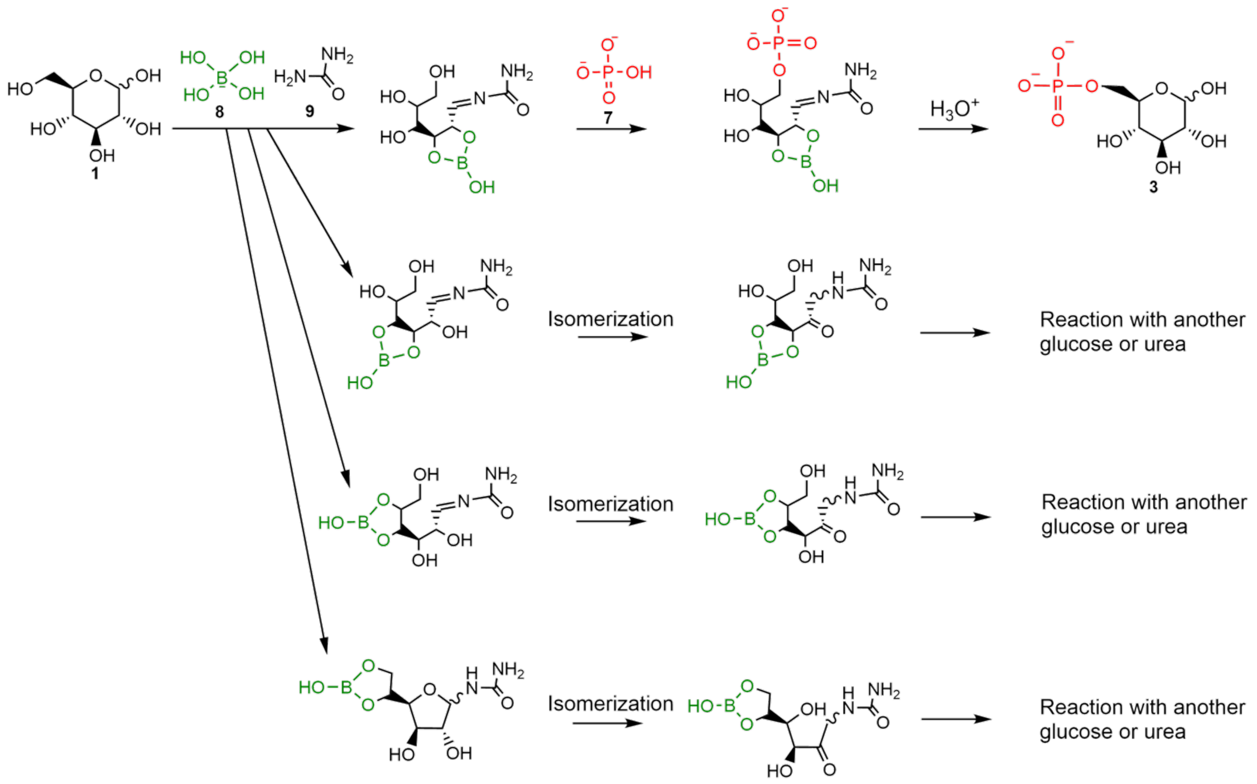


**Fig. S24. Possible reaction pathways from glucose.** Combining with borate at 2-hydroxyl, the isomerization from imine to ketone was prevented, which contributes to the stabilization of ureido-glucose. The stabilized molecules are subjected to the phosphorylation.

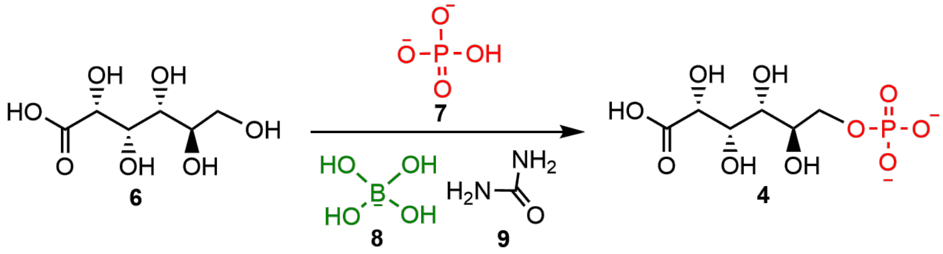


**Fig. S25. Possible gluconate phosphorylation pathway.**

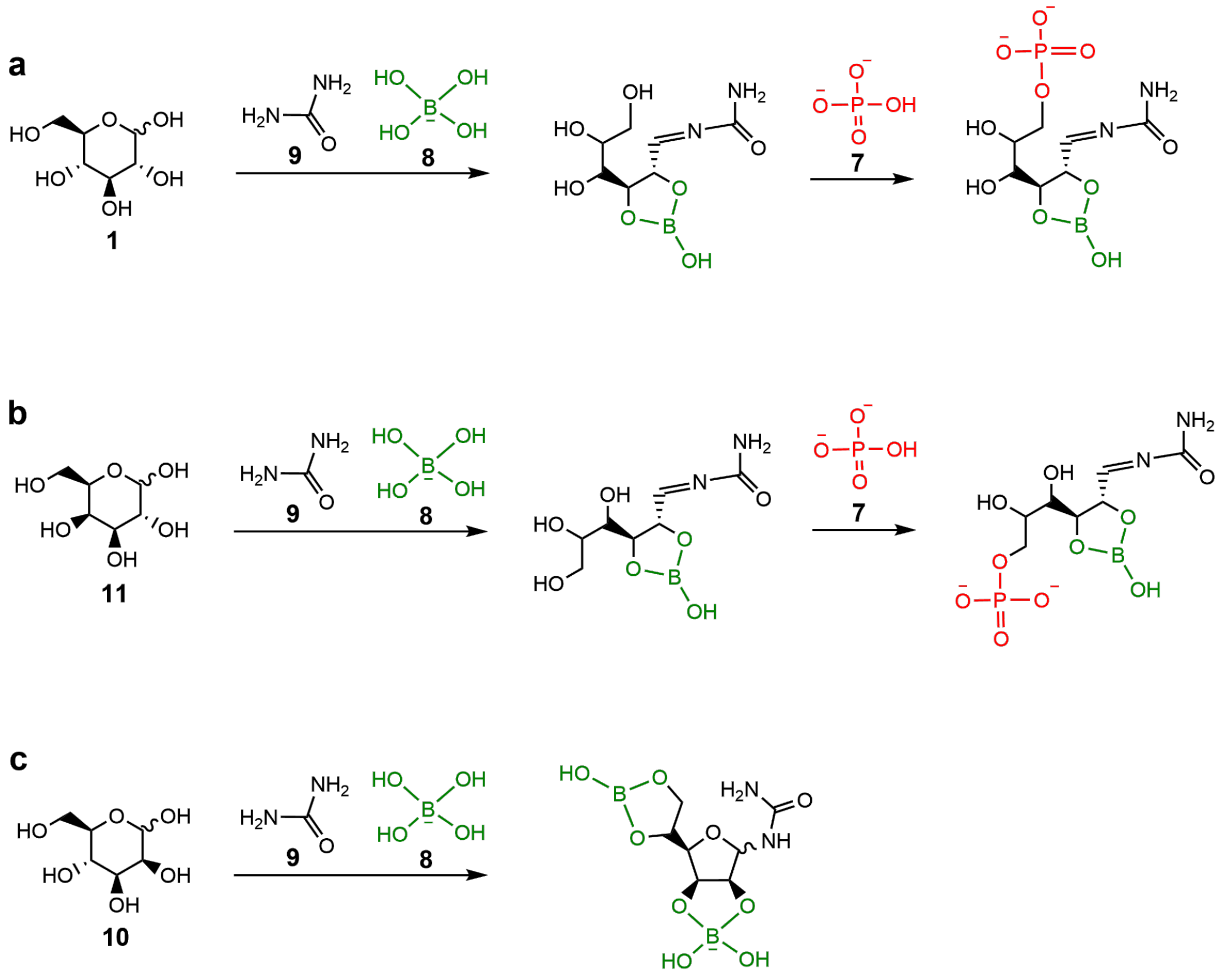


**Fig. S26. Possible phosphorylation pathways.** The structure of ureido hexoses is an example of such a possible structure. **(a)** The reaction pathways from glucose. **(b)** The reaction pathway from galactose. **(c)** The reaction pathway from mannose.

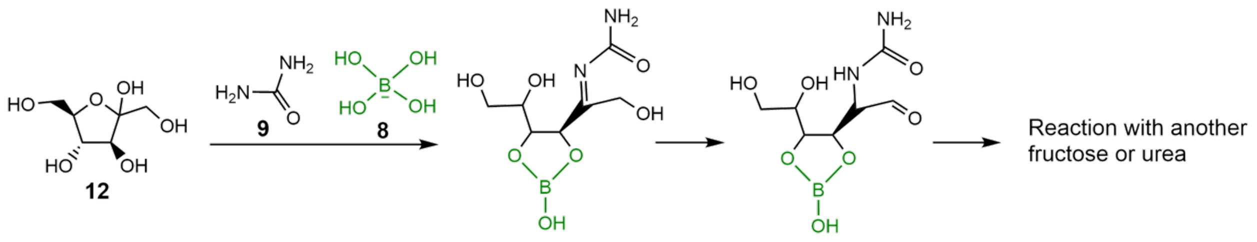


**Fig. S27. Possible reaction pathway from fructose.**

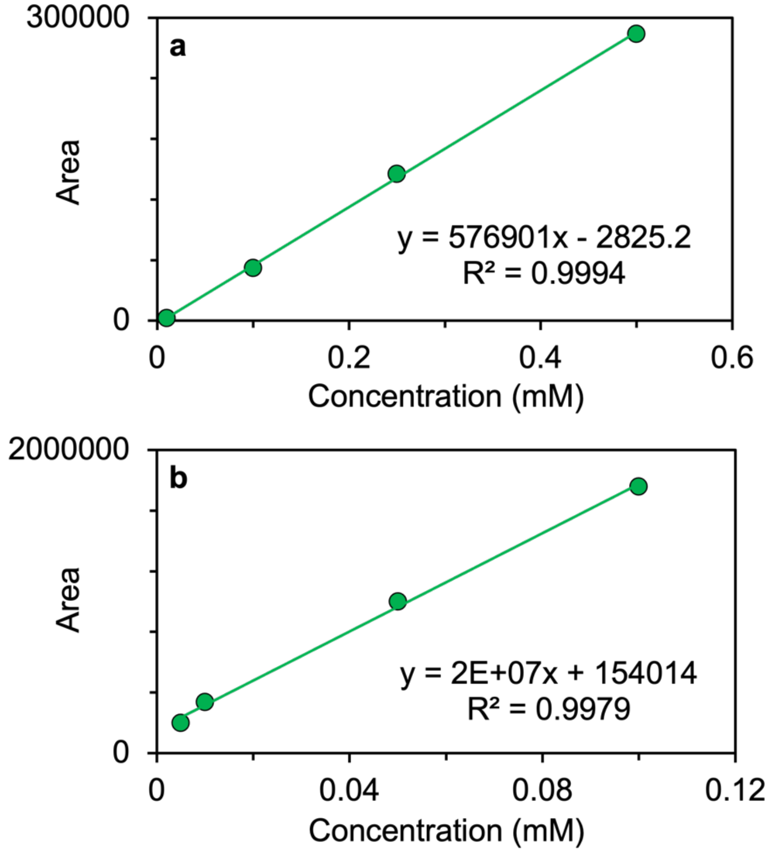


**Fig. S28. Calibration curves for the quantification of formose-like reaction products. (a)** Glucose **1** calibration curve. **(b)** gluconate **6** calibration curve.

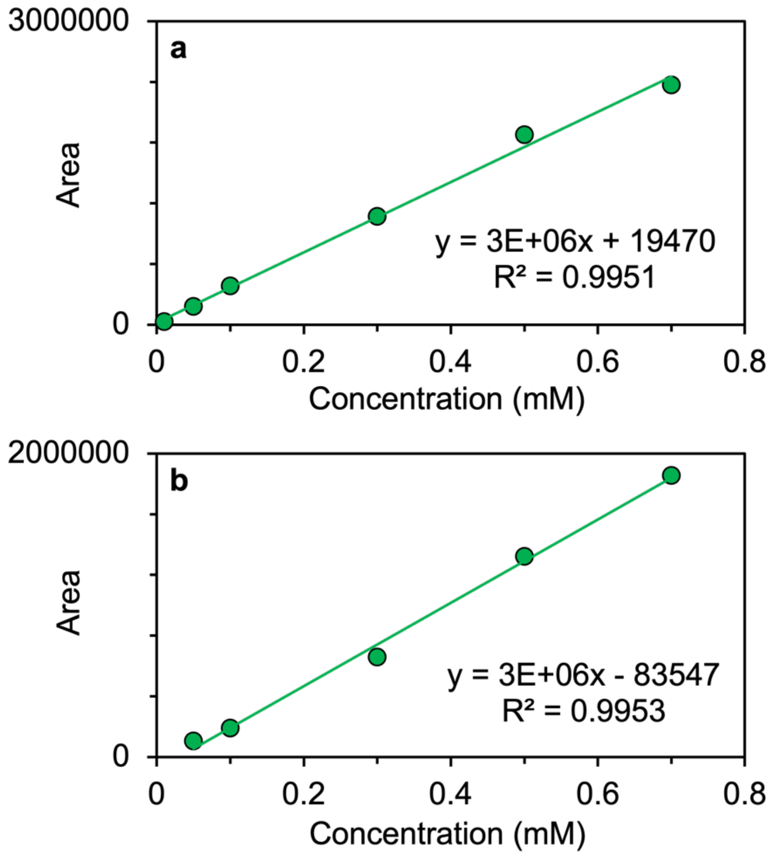


**Fig. S29. Calibration curves for the quantification of phosphorylation products. (a)** glucose 6-phosphate **3** calibration curve. **(b)** 6-phosphogluconate **4** calibration curve.

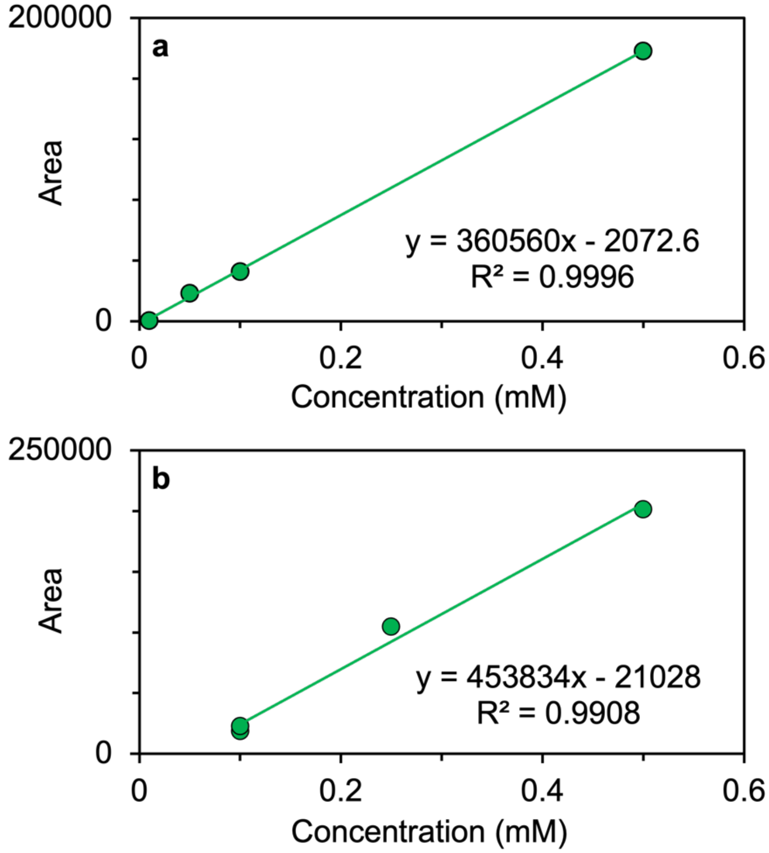


**Fig. S30. Calibration curves for the quantification of residual glucose 1 and gluconate 6. (a)** glucose **1** calibration curve. **(b)** gluconate **6** calibration curve.

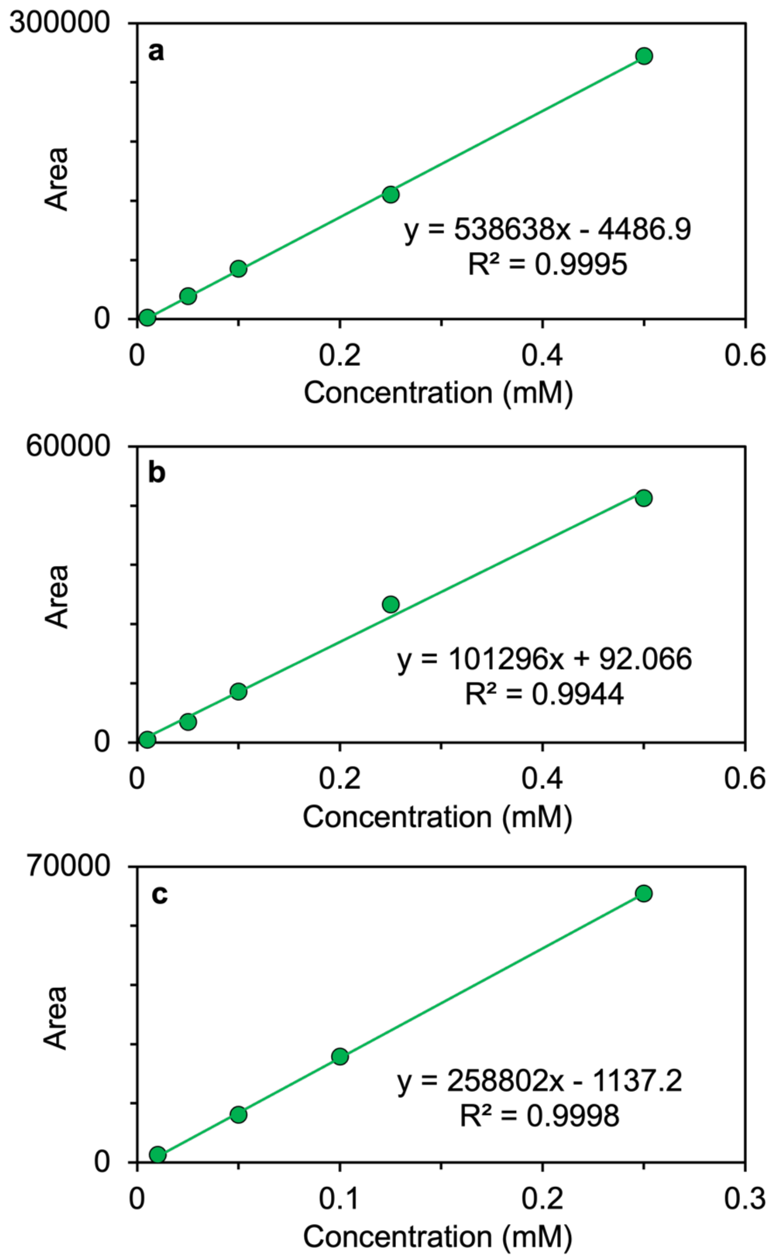


**Fig. S31. Calibration curves for the quantification of residual hexoses. (a)** Mannose **10** calibration curve. **(b)** galactose **11** calibration curve. **(c)** Fructose **12** calibration curve.
